# Selective Reduction of AMPA Currents onto Hippocampal Interneurons Impairs Network Oscillatory Activity

**DOI:** 10.1371/journal.pone.0037318

**Published:** 2012-06-04

**Authors:** Antonio Caputi, Elke C. Fuchs, Kevin Allen, Corentin Le Magueresse, Hannah Monyer

**Affiliations:** Department of Clinical Neurobiology, Medical Faculty of Heidelberg University and German Cancer Research Center (DKFZ), Heidelberg, Germany; Neuroscience Campus Amsterdam, VU University, Netherlands

## Abstract

Reduction of excitatory currents onto GABAergic interneurons in the forebrain results in impaired spatial working memory and altered oscillatory network patterns in the hippocampus. Whether this phenotype is caused by an alteration in hippocampal interneurons is not known because most studies employed genetic manipulations affecting several brain regions. Here we performed viral injections in genetically modified mice to ablate the GluA4 subunit of the AMPA receptor in the hippocampus (*GluA4^HC−/−^* mice), thereby selectively reducing AMPA receptor-mediated currents onto a subgroup of hippocampal interneurons expressing GluA4. This regionally selective manipulation led to a strong spatial working memory deficit while leaving reference memory unaffected. Ripples (125–250 Hz) in the CA1 region of *GluA4^HC−/−^* mice had larger amplitude, slower frequency and reduced rate of occurrence. These changes were associated with an increased firing rate of pyramidal cells during ripples. The spatial selectivity of hippocampal pyramidal cells was comparable to that of controls in many respects when assessed during open field exploration and zigzag maze running. However, GluA4 ablation caused altered modulation of firing rate by theta oscillations in both interneurons and pyramidal cells. Moreover, the correlation between the theta firing phase of pyramidal cells and position was weaker in *GluA4^HC−/−^* mice. These results establish the involvement of AMPA receptor-mediated currents onto hippocampal interneurons for ripples and theta oscillations, and highlight potential cellular and network alterations that could account for the altered working memory performance.

## Introduction

Network oscillatory patterns at different frequencies in the hippocampus represent distinct operating modes essential for normal spatial memory functions [1]. During exploration, theta oscillations (6–10 Hz) provide temporal windows for local circuit interactions important for the encoding and retrieval of spatial memories [2]–[7]. During immobility and slow-wave sleep, sharp wave/ripples (125–250 Hz, SWR) are associated with high levels of synchronous activity that could facilitate the stabilization of new memory traces and their consolidation into neocortical areas [1], [8]–[11].

The recruitment of GABAergic interneurons by pyramidal cells is thought to be crucial for these two network phenomena [12]–[17]. One experimental approach to causally link the recruitment of GABAergic interneurons to hippocampal functions has been to characterize mice in which excitatory currents onto interneurons have been modified. For example, mouse mutants with reduced AMPA or NMDA receptor-mediated currents only in parvalbumin-expressing interneurons exhibited altered network oscillations together with impaired spatial working memory [18]–[20]. However, because these manipulations affected interneurons in several brain regions, the phenotype could not unequivocally be linked to an alteration in hippocampal interneurons per se. In a recent study, Murray and colleagues [21] reported that blocking the synaptic output of parvalbumin-expressing hippocampal interneurons is sufficient to cause a severe spatial working memory impairment. However, the network alterations associated with such hippocampus-restricted manipulations are still unknown.

In addition to their involvement in network oscillations, hippocampal interneurons could also influence the spatial selectivity of hippocampal pyramidal cells. Recent modeling studies proposed that the spatial selectivity of hippocampal pyramidal cells is partially determined by the activity of local GABAergic interneurons [5], [22]–[28]. Moreover, hippocampal interneurons could control the progressive phase advancement of action potentials relative to theta oscillations [29]–[31], a phenomenon known as theta phase precession [7], [32].

The aim of this study was to investigate how a selective reduction of AMPA receptor-mediated currents onto hippocampal GABAergic interneurons affects hippocampal network oscillations and spatial coding. We targeted the AMPA receptor subunit GluA4, which is expressed exclusively in GABAergic interneurons in the hippocampus [19], [33], [34]. GluA4 ablation resulted in reduced AMPA receptor-mediated currents onto hippocampal interneurons but not onto pyramidal cells. This manipulation led to a spatial working memory impairment and alterations in SWRs and theta oscillations.

## Results

### Selective Ablation of GluA4 in Interneurons of the Dorsal Hippocampus

**Figure 1:**
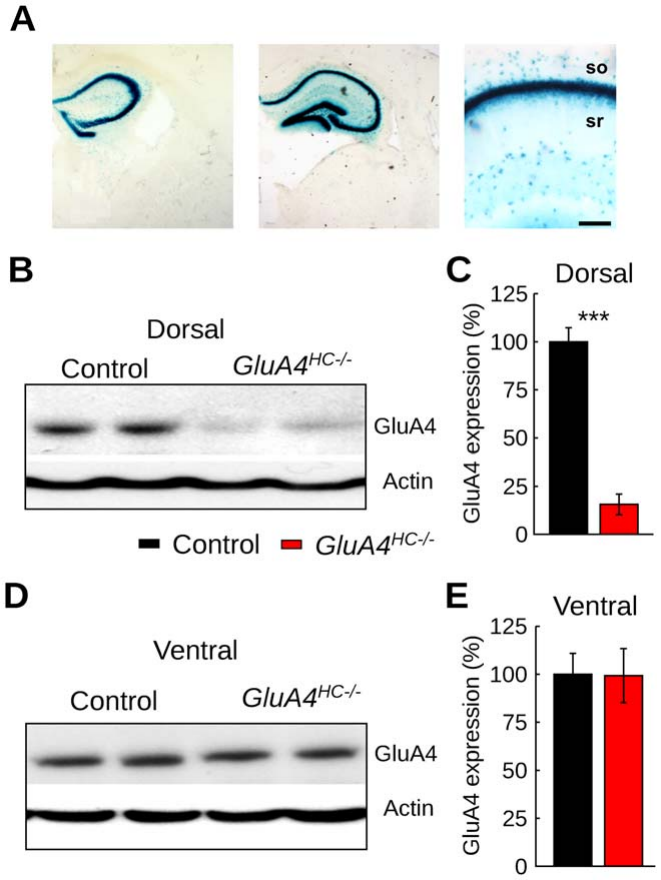
GluA4 ablation in the dorsal hippocampus. (A) Cre-mediated recombination of the *ROSA26* reporter gene after AAV-Cre injection into the dorsal hippocampus. Coronal sections at two anteroposterior levels of the hippocampus (left two panels) and a third section at higher magnification of the CA1 hippocampal region (right panel) showing pan-neuronal X-gal staining. Scale bar: 150 µm. (B) Representative Western blot from the dorsal hippocampus in control and *GluA4^HC−/−^* mice. (C) Quantification of GluA4 expression level in the dorsal hippocampus from Western blot analysis. Data are expressed as percentage of control levels (mean ± SEM). (D) Representative Western blot of the ventral hippocampus in control and *GluA4^HC−/−^* mice. (E) Quantification of GluA4 expression level in the ventral hippocampus from Western blot analysis. Abbreviations: so, stratum oriens; sr, stratum radiatum. ***: *p*<10^−10^.

Restricted hippocampal GluA4 ablation was achieved by bilateral injections of an adeno-associated virus expressing Cre recombinase (AAV-Cre) into the dorsal hippocampus of adult mice. The extent of recombination activity after bilateral AAV-Cre injections was established by injecting AAV-Cre into *ROSA26* reporter mice (*n* = 8 mice). Recombination was observed in the dorsal hippocampus (Figure 1A), but not in the ventral hippocampus or in surrounding brain areas. Close examination of histological material revealed that recombination took place both in principal cells and interneurons of the hippocampus.

We generated mice with dorsal hippocampal GluA4 ablation (*GluA4^HC−/−^*) by injecting AAV-Cre into *GluA4^2lox^* mice [19]. AAV-Cre-injected wildtype or AAV-tomato-injected *GluA4^2lox^* littermates served as controls throughout the study. GluA4 expression levels in the dorsal and ventral hippocampus were analyzed by Western blot. There was a significant decrease of GluA4 expression in the dorsal hippocampus (Figure 1B and C, control *n* = 12 mice, *GluA4^HC−/−^ n* = 12 mice, *p*<10*^−^* ^10^), but not in the ventral hippocampus (Figure 1D and E, *p*>0.5) or in extra-hippocampal regions surrounding the injection site (Figure S1A *−*B). GluA4 ablation did not affect the expression of other glutamate receptor subunits (Figure S1C−D) and did not lead to visible alterations in hippocampal anatomy or parvalbumin expression (Figure S2).

**Figure 2:**
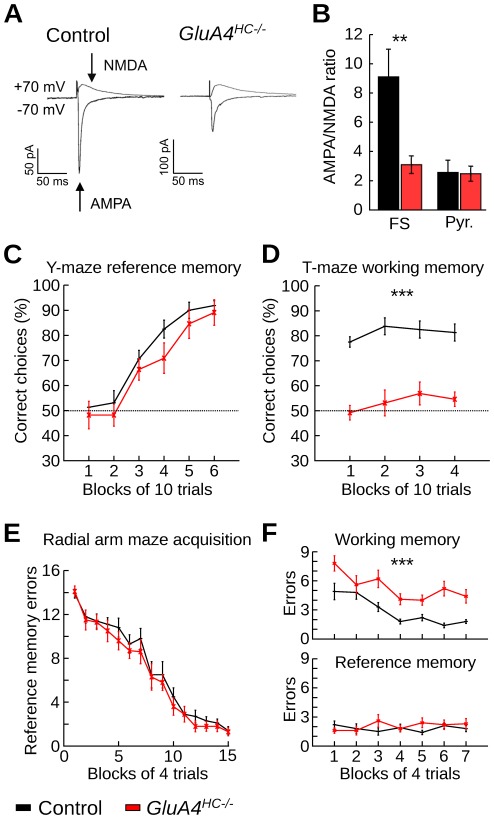
GluA4 deletion affects the recruitment of interneurons and spatial working memory. (A) Representative averaged excitatory postsynaptic currents (EPSCs) from a fast-spiking interneuron in control and *GluA4^HC−/−^* mice. Arrows indicate the time points at which the AMPA and NMDA components were measured. Traces in control and *GluA4^HC−/−^* mice were normalized to the size of the EPSC measured at +70 mV. (B) AMPA/NMDA ratio in fast-spiking cells and pyramidal cells of control and *GluA4^HC−/−^* mice. (C) Percentage of correct choices during the acquisition of an appetitive hippocampus-dependent spatial reference memory task on an elevated Y-maze in control and *GluA4^HC−/−^* mice. The dotted line represents chance levels. (D) Percentage of correct choices during hippocampus-dependent rewarded alternation task on the elevated T-maze for control and *GluA4^HC−/−^* mice. (E) Reference memory errors per block (maximum of 16 errors) for control and *GluA4^HC−/−^* mice during reference memory acquisition of the radial arm maze task (doors prevented working memory errors). (F) Number of working memory (top) and reference memory (bottom) errors during simultaneous assessment of working and reference memory on the radial arm maze. Abbreviations: FS, fast-spiking interneurons; Pyr., pyramidal cells. **: *p*<0.01, ***: *p*<10*^−^* ^11^.

We obtained functional evidence for selective GluA4 deletion in GABAergic interneurons of the hippocampus by performing whole cell patch-clamp recordings in CA1 pyramidal cells and fast-spiking putative parvalbumin-expressing interneurons (Table S1). These interneurons were assessed because they account for approximately 80% of hippocampal GluA4-expressing neurons [19]. There was a reduction in evoked synaptic AMPA receptor-mediated currents of fast-spiking cells in *GluA4^HC−/−^* mice compared to controls (Figure 2A and B, control: 17 cells from 9 mice, *GluA4^HC−/−^*: 14 cells from 9 mice, difference in AMPA/NMDA ratio: *p*<0.01). Residual AMPA receptor-mediated currents in *GluA4^HC−/−^* fast-spiking cells can be attributed to the expression of other AMPA receptor subunits, in particular GluA1 [34]. The AMPA/NMDA ratio of pyramidal cells in control and *GluA4^HC−/−^* mice was not significantly different (Figure 2B, control *n* = 16 cells, *GluA4^HC−/−^ n* = 14 cells, *p*>0.5). Further evidence for the cell type-specific GluA4 ablation was provided by the differential effect on AMPA receptor decay time in fast-spiking and pyramidal cells. It was previously established that GluA4 expression confers fast decay kinetics to AMPA receptors [34]. Accordingly, in *GluA4^HC−/−^* mice the decay time of evoked AMPA receptor-mediated excitatory postsynaptic currents was slower in fast-spiking cells but was not affected in pyramidal neurons (interneurons: control *n* = 17 cells, *GluA4^HC−/−^ n* = 14 cells, control 4.22±0.41 ms, *GluA4^HC−/−^*6.74±1.10 ms, *p*<0.05; pyramidal cells: control *n* = 16 cells, *GluA4^HC−/−^ n* = 14 cells, control 11.1±0.8 ms, *GluA4^HC−/−^*12.7±1.0 ms, *p*>0.1). As expected, the decay time of evoked NMDA receptor-mediated excitatory postsynaptic currents was unaffected in fast-spiking cells (control *n* = 17 cells, *GluA4^HC−/−^ n* = 14 cells, control 63.2±5.9 ms, GluA4^HC*−*/*−*^65.4±5.1 ms, *p*>0.5).

At the behavioral level, *GluA4^HC−/−^* mice displayed normal spatial reference memory on the Y-maze (Figure 2C, control *n* = 16 mice, *GluA4^HC−/−^ n* = 13 mice, main effect of genotype, *F*_(1,27)_ = 3.2, *p*>0.05). However, they were severely impaired on the rewarded alternation task on the T-maze (Figure 2D, control *n* = 16 mice, *GluA4^HC−/−^ n* = 13 mice, main effect of genotype, *F*_(1,27)_ = 122.6, *p*<10*^−^* ^18^), a hippocampus-dependent task sensitive to alteration in interneuron activity [19], [20], [35]. Furthermore, the mice were tested on an eight-arm radial maze. After successfully acquiring reference memory on the 4-out-of-8 version of the radial arm maze (Figure 2E, control *n* = 16 mice, *GluA4^HC−/−^ n* = 12 mice, control: 1.4±0.4 errors, *GluA4^HC−/−^*: 1.3±0.4 errors, main effect of *genotype*, *F*_(1,26)_ = 3.0, *p*>0.05), *GluA4^HC−/−^* mice made more working memory errors when they were no longer prevented from re-entering a previously visited arm within a trial (Figure 2F, control 2.9±0.2 errors; *GluA4^HC−/−^*5.3±0.2 errors, *F*_(1,26)_ = 56.6, *p*<10*^−^* ^11^). In mice that underwent behavioral testing, hippocampal GluA4 deletion was subsequently confirmed by Western blot analysis (Figure S1C). This demonstrates that GluA4 ablation had significant consequences at the behavioral level. Moreover, the observed behavioral impairments are consistent with those obtained in other mouse mutants in which the activity of interneurons was affected [19], [21].

### *In vivo* Recordings from the CA1 Region of *GluA4^HC−/−^* Mice

Cell activity and network oscillations from the CA1 pyramidal cell layer were recorded in control and *GluA4^HC−/−^* mice (control: 440 neurons from 9 mice, *GluA4^HC−/−^*: 373 neurons from 12 mice, Table S2). A total of 29 and 37 recording sessions were analyzed in control and mutant mice, respectively. Manually refined spike clusters were automatically classified as pyramidal cells or interneurons based on their mean firing rate, spike-time autocorrelation and spike duration (Figure S3A *-*D) [15]. The proportion of cells classified as pyramidal cells and interneurons did not differ across genotypes (control: 362 pyramidal cells and 65 interneurons, *GluA4^HC−/−^*: 324 pyramidal cells and 41 interneurons, χ^2^_(1)_ = 2.37, *p* = 0.12). There was no difference in the quality of cluster isolation for pyramidal cells recorded in control and *GluA4^HC−/−^* mice (Figure S3E, control *n* = 362 cells, *GluA4^HC−/−^ n* = 324 cells, *p* = 0.33). In addition, the mean waveforms of pyramidal cell or interneuron spikes were similar across genotypes (Figure S3F). At the behavioral level, there was no difference in running speed between control and *GluA4^HC−/−^* mice in the three environments used during the recording sessions (Figure S4, control *n* = 29 sessions, *GluA4^HC−/−^ n* = 37 sessions, rest cage: *p* = 0.26, open field: *p* = 0.23, zigzag maze: *p* = 0.37).

### Altered SHARP Wave/Ripples in *GluA4^HC−/−^* Mice

**Figure 3:**
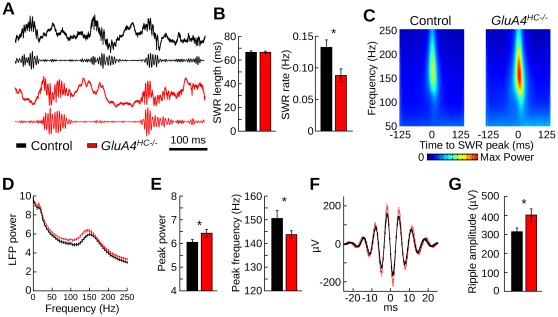
Local field potentials during sharp wave/ripples in *GluA4^HC−/−^* mice. (A) Representative examples of SWRs recorded during a rest trial in a control and a *GluA4^HC−/−^* mouse. Top trace: raw signal. Bottom trace: band-pass filtered (125–250 Hz) signal. (B) Mean length of SWR epochs and frequency of SWR occurrence in control and *GluA4^HC−/−^* mice. (C) Mean time-frequency representation of power centered on the peak power of each SWR epoch. (D) Mean power spectrum of SWRs in control and *GluA4^HC−/−^* mice. (E) Mean peak ripple frequency and mean peak power during SWRs in control and *GluA4^HC−/−^* mice. (F) Mean waveform of ripples centered on the peak power of each SWR epoch and aligned on the positive-to-negative zero-crossing. (G) Mean ripple amplitude in control and *GluA4^HC−/−^* mice. *: *p*<0.05.

SWRs were observed principally during periods of immobility. Examples are shown in Figure 3A. The duration of SWRs was similar across genotypes (Figure 3B, control *n* = 9 mice, *GluA4^HC−/−^ n* = 12 mice, *p* = 0.98), but they occurred less frequently in *GluA4^HC−/−^* mice compared to controls (Figure 3B, control *n* = 9 mice, *GluA4^HC−/−^ n* = 12 mice, *p* = 0.015). We calculated a mean time-frequency representation of power around SWR peak power for each control and *GluA4^HC−/−^* mouse (Figure 3C). The peak power between 125 and 250 Hz was higher in *GluA4^HC−/−^* mice compared to control mice. The mean power spectrum calculated during SWRs also showed that the peak ripple power was higher in *GluA4^HC−/−^* mice (Figure 3D–E, control *n* = 9 mice, *GluA4^HC−/−^ n* = 12 mice, *p* = 0.01). Moreover, the peak power occurred at slower frequency in *GluA4^HC−/−^* mice compared to control mice (Figure 3D–E, *p* = 0.04). The mean SWR waveform centered on the peak SWR power also indicated larger ripple amplitude in *GluA4^HC−/−^* mice (Figure 3F–G, control *n* = 9 mice, *GluA4^HC−/−^ n* = 12 mice, *p* = 0.01).

**Figure 4:**
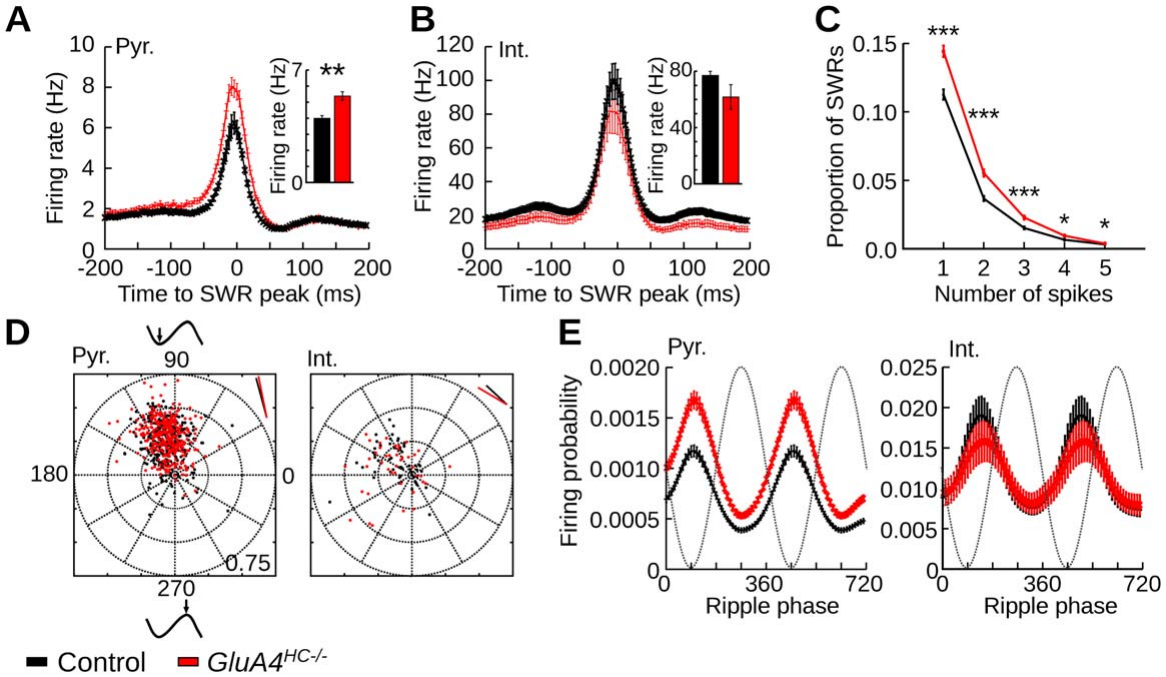
Cell activity during sharp wave/ripples in *GluA4^HC−/−^* mice. (A) Firing rate of pyramidal cells during SWRs. Time 0 represents the peak power of SWRs. The inset shows the mean firing rate during SWRs. (B) Same as A but for interneurons. (C) Proportion of SWRs in which a pyramidal fire from 0 to 5 spikes. Pyramidal cells in *GluA4^HC−/−^* mice were more likely than that of control mice to fire between 1 to 5 spikes during a SWR. (D) Polar plot of the preferred ripple phase and ripple vector length of pyramidal cells (left) and interneurons (right) in control and *GluA4^HC−/−^* mice. Each dot represents a neuron. Phase 0 is the positive-to-negative zero-crossing of the ripple. The ripple vector length of each cells is equal to the distance between the dot and the the center of the plot. The short lines in the right-top corner indicate the mean preferred phase of the recorded neurons. (E) Mean firing probability at different ripple phases for pyramidal cells (left) and interneurons (right) in control and *GluA4^HC−/−^* mice. Abbreviations: Int., interneurons; Pyr., pyramidal cells. **: *p*<0.01, ***: *p*<10*^−^* ^5^.

These changes in SWR occurrence, peak power and peak frequency were accompanied by an increased firing rate in pyramidal cells of *GluA4^HC−/−^* mice during SWRs (Figure 4A, Figure S5A, control *n* = 362 cells, *GluA4^HC−/−^ n* = 324 cells, control: 3.98±0.179 Hz, *GluA4^HC−/−^*: 5.38±0.259 Hz, *p*<10*^−^* ^5^). The firing rate of interneurons during SWRs in *GluA4^HC−/−^* mice was not significantly different from that in control mice (Figure 4B, control *n* = 65 cells, *GluA4^HC−/−^ n* = 41 cells, control: 77.01±7.82 Hz, *GluA4^HC−/−^*: 61.81±8.76 Hz, *p* = 0.23). We calculated the probability that a pyramidal cell fires 0 to 5 spikes during a SWR (Figure 4C). Pyramidal cells in mutant mice were less likely to be silent during SWRs (control *n* = 362 cells, *GluA4^HC−/−^ n* = 324 cells, probability of being silent, control: 0.83±0.006, *GluA4^HC−/−^*: 0.78±0.008, *p*<10*^−^* ^5^) and more likely to fire between 1 and 5 spikes (Figure 4C, 1 spike, *p*<10*^−^* ^5^; 2 spikes, *p*<10*^−^* ^6^; 3 spikes, *p*<10*^−^* ^5^; 4 spikes, *p* = 0.0007; 5 spikes, *p* = 0.0003). The same analysis performed on interneurons did not reveal any significant difference between the two genotypes (Figure S5C, control *n* = 65 cells, *GluA4^HC−/−^ n* = 41 cells, *p*>0.05).

The firing of most pyramidal cells and interneurons was significantly modulated by ripples in both genotypes (pyramidal cells, control *n* = 362 cells, *GluA4^HC−/−^ n* = 324 cells, control: 78.70%, *GluA4^HC−/−^*: 81.20%, difference between groups: *p* = 0.52, interneurons, control *n* = 65 cells, *GluA4^HC−/−^ n* = 41 cells, control: 77.19%, *GluA4^HC−/−^*: 80.56%, difference between groups: *p* = 0.90). The preferred firing ripple phase of pyramidal cells and their ripple vector length were not significantly different across genotypes (Fig 4*D*, control *n* = 362 cells, *GluA4^HC−/−^ n* = 324 cells, preferred phase, control: 103.54°, *GluA4^HC−/−^*: 102.35°, *p*>0.10, mean vector length, control: 0.309±0.0074, *GluA4^HC−/−^*: 0.309±0.0097, *p* = 0.848). In addition, we found no difference in the preferred firing phase and mean vector length of interneurons (Figure 4D, control *n* = 65 cells, *GluA4^HC−/−^ n* = 41 cells, preferred phase, control: 150.24°, *GluA4^HC−/−^*: 152.56°, *p*>0.10, mean vector length, control: 0.217±0.018, *GluA4^HC−/−^*: 0.251±0.023, *p* = 0.20). As expected, the mean firing probability of pyramidal cells during ripples was higher in *GluA4^HC−/−^* mice than that in controls (Figure 4E, control *n* = 362 cells, *GluA4^HC−/−^ n* = 324 cells, difference in firing probability, 90°: *p*<10*^−^* ^7^, 180°: *p*<10*^−^* ^6^, 270°: *p* = 0.0013, 360°: *p*<10*^−^* ^7^). The mean firing probability of interneurons during ripples was not significantly different across genotypes (Figure 4E, control *n* = 65 cells, *GluA4^HC−/−^ n* = 41 cells, all *p* values >0.25).

### Altered Firing During Theta Oscillations in *GluA4^HC−/−^* Mice

**Figure 5:**
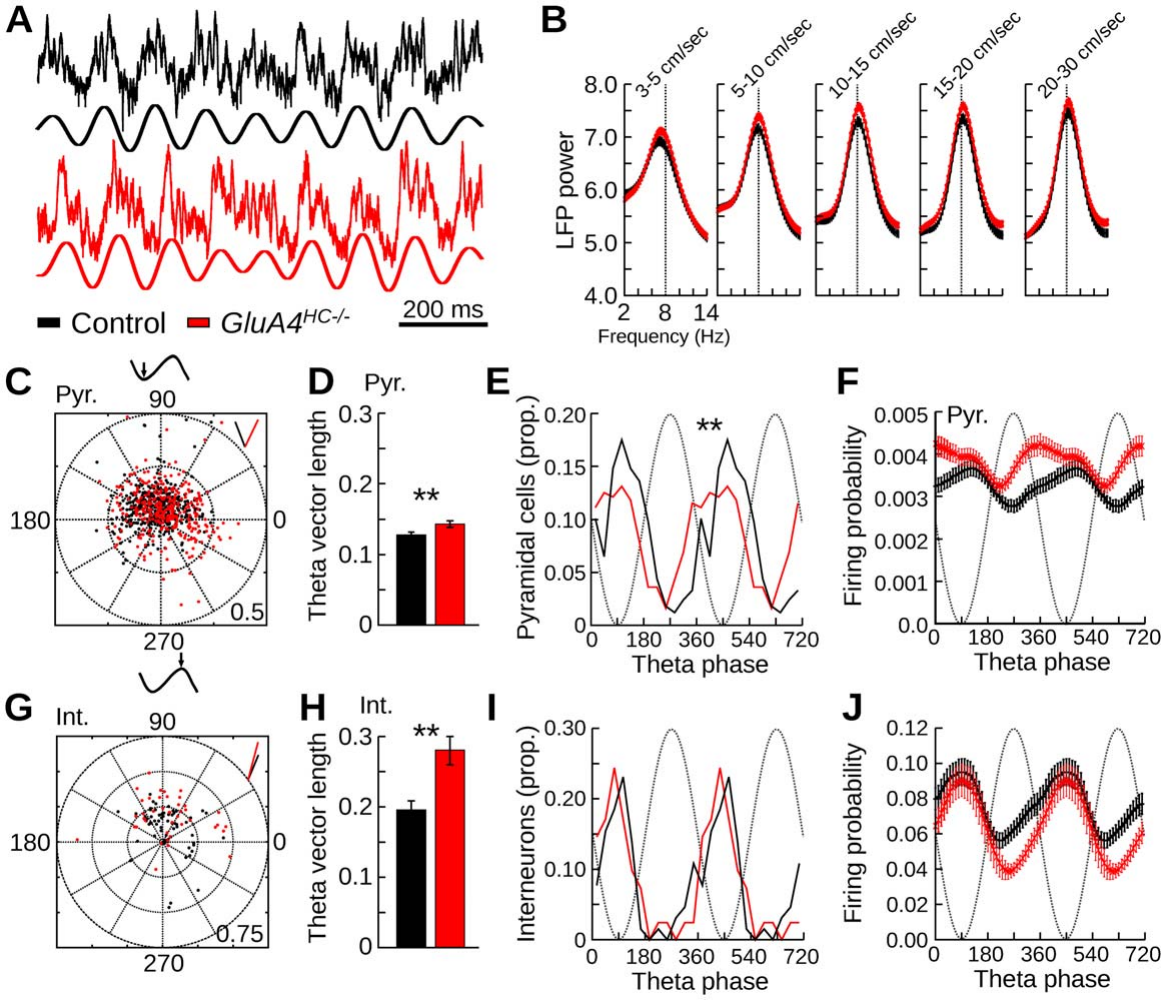
Theta oscillations in *GluA4^HC−/−^* mice. (A) Representative examples of theta oscillations recorded during exploratory trials in control and *GluA4^HC−/−^* mice. Top trace: raw signal. Bottom trace: band-pass filtered (5–14 Hz) signal. (B) Mean power spectra in the theta frequency range when mice ran at different speed. The peak power and peak frequency was similar in the control and *GluA4^HC−/−^* mice. (C) Polar plot of the preferred theta phase and theta vector length of pyramidal cells in control and *GluA4^HC−/−^* mice. Each dot represents a neuron. Phase 0 is the positive-to-negative zero-crossing of the theta oscillation. The theta vector length of each cells is equal to the distance between the dot and the center of the plot. The short lines in the right-top corner indicate the mean preferred phase of the recorded neurons. (D) Mean theta vector length for all pyramidal cells in control and *GluA4^HC−/−^* mice. (E) Distribution of preferred theta phase for pyramidal cells in control and *GluA4^HC−/−^* mice. (F) Mean firing probability at different theta phases for pyramidal cells. (G–J) Same as C–F but for interneurons. Abbreviations: Int., interneurons; Pyr., pyramidal cells. **: *p*<0.005.

Prominent theta oscillations were recorded from the CA1 pyramidal cell layer in control and *GluA4^HC−/−^* mice (Figure 5A). Power spectra were calculated as the mice ran at different speed intervals during exploratory trials (Figure 5B). The peak theta power or the mean peak theta frequency of the power spectra were not changed in *GluA4^HC−/−^* mice (Figure 5B and Figure S6, control *n* = 9 mice, *GluA4^HC−/−^ n* = 12 mice), suggesting that the frequency and power of theta oscillations were normal in *GluA4^HC−/−^* mice. The burst theta frequency of pyramidal cells and interneurons, as assessed by their spike-time autocorrelation during theta epochs, was also similar in the two genotypes (Figure S6C–D, pyramidal cells, control *n* = 362 cells, *GluA4^HC−/−^ n* = 324 cells, control: 115.16±0.70 ms, *GluA4^HC−/−^*: 114.83±0.65 ms, *p* = 0.16; interneurons, control *n* = 65 cells, *GluA4^HC−/−^ n* = 41 cells, control: 113.22±1.99 ms, *GluA4^HC−/−^*: 117.83±1.73 ms, *p* = 0.40).

We investigated whether reduced AMPA currents onto hippocampal interneurons affect the temporal firing properties of neurons relative to theta oscillations. The firing of 88.4 and 94.14% of pyramidal cells in control and *GluA4^HC−/−^* mice, respectively, was modulated by theta oscillations (control *n* = 362 cells, *GluA4^HC−/−^ n* = 324 cells, Rayleigh test with *p*<0.01, difference between groups: *p* = 0.012). The preferred theta phase and the theta vector length of individual pyramidal cells are shown in Figure 5C. Pyramidal cells in *GluA4^HC−/−^* mice tended to be more theta modulated than those of control mice (Figure 5D, control *n* = 362 cells, *GluA4^HC−/−^ n* = 324 cells, *p* = 0.0029). Moreover, the preferred theta phase of pyramidal cells occurred at an earlier phase in *GluA4^HC−/−^* mice (Figure 5E, control *n* = 362 cells, *GluA4^HC−/−^ n* = 324 cells, mean preferred theta phase, control: 111.65°, *GluA4^HC−/−^*: 66.03°, *p*<0.001). Significantly more pyramidal cells in *GluA4^HC−/−^* mice had a preferred phase at the descending portion of the theta cycle. Accordingly, the mean firing probability of pyramidal cells in *GluA4^HC−/−^* mice was higher than that of controls mainly at the peak and the early descending phase of theta oscillations (Figure 5F, control *n* = 362 cells, *GluA4^HC−/−^ n* = 324 cells, difference in firing probability, 90°: *p* = 0.0084, 180°: *p* = 0.048, 270°: *p*<10*^−^* ^5^, 360°: *p*<10*^−^* ^5^). The alteration in preferred theta firing phase in *GluA4^HC−/−^* mice was present when the mice ran in the open field or in the zigzag maze (*p*<0.001).

The firing rate of all but one interneuron in a control mouse was significantly modulated by theta oscillations. The preferred phase and vector length of each interneuron is shown in Figure 5G. The mean theta vector length was significantly longer in *GluA4^HC−/−^* mice than in control mice (Figure 5H, control *n* = 65 cells, *GluA4^HC−/−^ n* = 41 cells, *p* = 0.00018). This difference was observed in both the open field and the zigzag maze (*p*<0.001). In contrast to pyramidal cells, the distribution of preferred theta firing phase for interneurons was not significantly different across genotypes (Figure 5I, control *n* = 65 cells, *GluA4^HC−/−^ n* = 41 cells, mean preferred theta phase, control: 67.48°, *GluA4^HC−/−^*: 75.12°, *p*>0.10). The mean firing probability of interneurons in *GluA4^HC−/−^* mice was lower than that of control mice at the peak and early descending phase of theta oscillations (Figure 5J, control *n* = 65 cells, *GluA4^HC−/−^ n* = 41 cells, difference in firing probability, 90°: *p* = 0.66, 180°: *p* = 0.097, 270°: *p* = 0.00073, 360°: *p* = 0.12 ).

### Spatial Selectivity of Place Cells in *GluA4^HC−/−^* Mice During Open-Field Exploration

**Figure 6:**
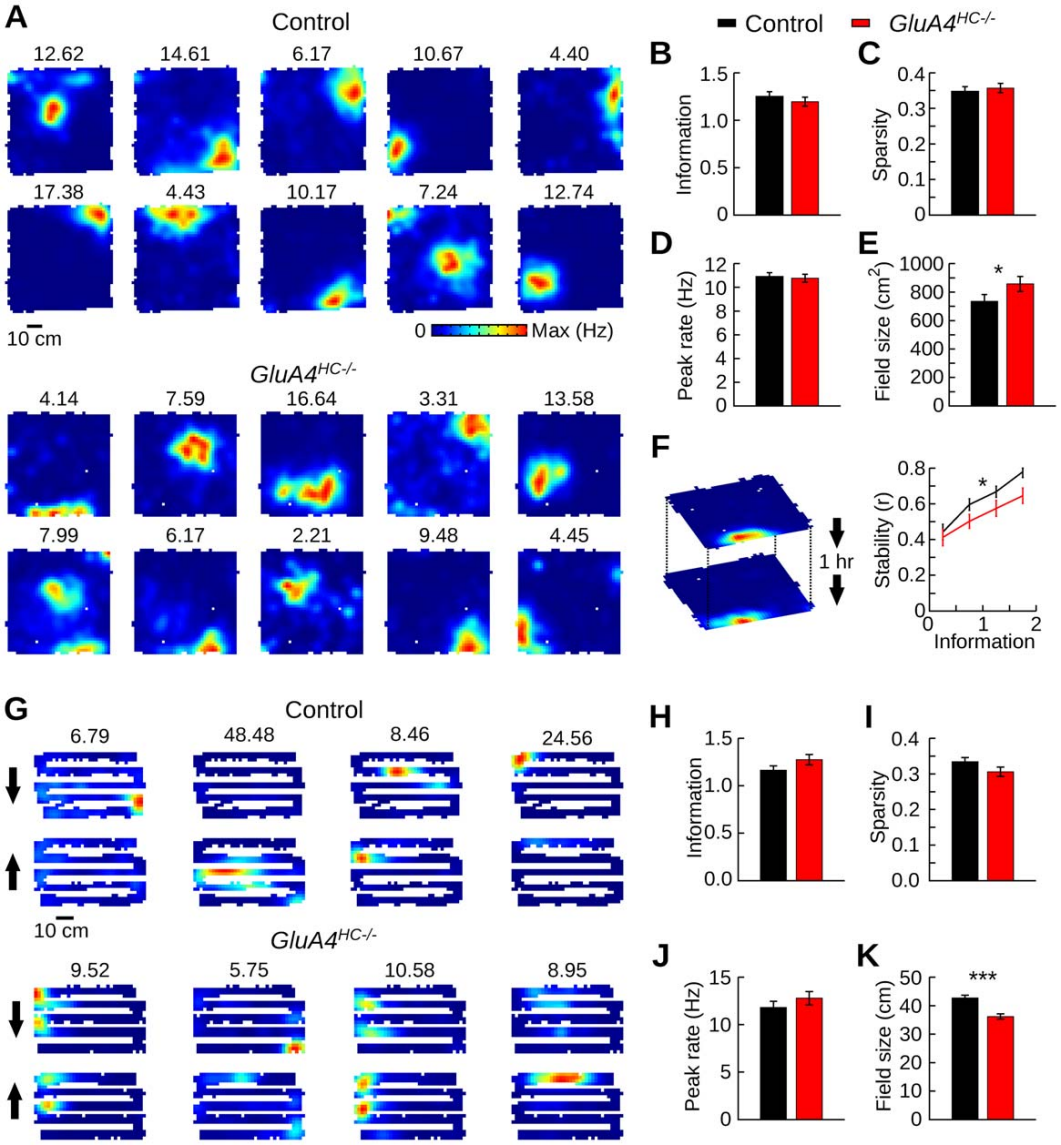
Spatial firing during trials in the open field and the zigzag maze. (A) Representative examples of firing rate maps in the open field from 10 simultaneously recorded pyramidal cells in a control and a *GluA4^HC−/−^* mouse. Numbers above each map indicate the peak firing rate in Hz. (B) Spatial information score in the open field for pyramidal cells in control and *GluA4^HC−/−^* mice (mean ± SEM). (C) Sparsity score in the open field for pyramidal cells in control and *GluA4^HC−/−^* mice. (D) Peak firing rate of firing fields in the open field. (E) Mean place field size in the open field. (F) Stability of place firing rate maps across two trials in the open field. Representative recording of one cell during two trials in the open field (1 hr inter-trial interval, left panel). Stability of place firing rate maps with different spatial information scores (right panel). (G) Representative examples of firing rate maps in the zigzag maze from 4 pyramidal cells recorded in a control and a *GluA4^HC−/−^* mouse. South- and northbound runs (indicated by arrows) are plotted separately (top and bottom rows). Numbers above each map indicate the peak firing rate in Hz. (H) Spatial information score in the zigzag maze for pyramidal cells in the zigzag maze. (I) Sparsity score in the zigzag maze for pyramidal cells. (J) Peak firing rate in the 2-dimensional firing rate maps in the zigzag maze for pyramidal cells of control and *GluA4^HC−/−^* mice. (K) Mean size of the firing fields detected in 1-dimensional firing rate maps of the zigzag maze. *: *p*<0.05, ***: *p*<10*^−^* ^7^.

We tested whether hippocampal GluA4 ablation affects the degree of spatial selectivity of hippocampal place cells. Spatial firing in the open field was assessed in active pyramidal cells (>300 spikes) during the first daily exploratory trial in this environment. Figure 6A shows the firing rate maps of 10 pyramidal cells recorded during a single recording session in a control and a *GluA4^HC−/−^* mouse. One or more locations of high firing could be observed in most active pyramidal cells of both genotypes. There was no significant change in the spatial information score (Figure 6B, control *n* = 244 cells, *GluA4^HC−/−^ n* = 229 cells, *p* = 0.99) or spatial sparsity score (Figure 6C, *p*>0.80), indicating that pyramidal cells in *GluA4^HC−/−^* mice retained a normal degree of spatial selectivity. Active pyramidal cells of control and *GluA4^HC−/−^* mice had on average 0.96±0.06 and 0.96±0.05 place fields, respectively (control *n* = 244 cells, *GluA4^HC−/−^ n* = 229 cells, *p* = 0.48). There was no difference in the peak firing rate of the detected fields (Figure 6D, control *n* = 235 fields, *GluA4^HC−/−^ n* = 220 fields, *p* = 0.32), but the field size in *GluA4^HC−/−^* mice was slightly larger than that in control mice (Figure 6E, *p* = 0.017). This relatively weak effect disappeared when the mean place field size was calculated for each mouse and the statistical analysis performed on the two groups of mice (Figure S7A, control *n* = 8 mice, *GluA4^HC−/−^ n* = 12 mice, *p*>0.20). This suggests that there was no systematic increase of place field size in *GluA4^HC−/−^* mice during open-field exploration. The same conclusion was reached when the analysis was performed on the second daily trial in the open field (Figure S7B, control *n* = 8 mice, *GluA4^HC−/−^ n* = 12 mice, *p*>0.20).

The stability of the firing rate maps across two 20-min foraging trials in the open field separated by a 1 h inter-trial interval was quantified by calculating a Pearson correlation coefficient between the firing rate values of the maps of the two trials (Figure 6F). The stability of the firing maps was lower in *GluA4^HC−/−^* mice than in control mice (control *n* = 283 cells, *GluA4^HC−/−^ n* = 263 cells, *p* = 0.00038) and firing rate map stability was positively correlated with information score (*n* = 546 cells, *r* = 0.33, *p*<10*^−^* ^15^). Taken together, these results indicate that although pyramidal cells in *GluA4^HC−/−^* mice expressed a degree of spatial selectivity close to that of control, the stability of their spatial representation across trials was reduced.

### Place Cell Spatial Selectivity in *GluA4^HC−/−^* Mice During Zigzag Maze Running

The spatial selectivity of hippocampal pyramidal cells was then assessed in a zigzag maze. The multicompartment environment comprised 5 alleys with similar geometrical features. We treated the southbound and northbound runs as non-overlapping locations, because the spatial firing of place cells varies on the two types of journeys [36], [37]. Figure 6G shows the firing rate maps of 4 simultaneously recorded pyramidal cells in both genotypes. The spatial information and spatial sparsity scores were calculated from the 2-dimensional firing rate maps. Spatial selectivity, as estimated from these two scores, was normal in *GluA4^HC−/−^* mice (Figure 6H–I, control *n* = 213 cells, *GluA4^HC−/−^ n* = 174 cells, information score: *p* = 0.092, sparsity score: *p* = 0.123). The peak firing rates in 2-dimensional firing rate maps were also normal in *GluA4^HC−/−^* mice (Figure 6J, control *n* = 213 cells, *GluA4^HC−/−^ n* = 174 cells, *p* = 0.079). The firing fields of pyramidal cells were then detected in one-dimensional firing maps of the zigzag maze. Pyramidal cells in *GluA4^HC−/−^* mice had more firing fields on the zigzag maze (control *n* = 213 cells, *GluA4^HC−/−^ n* = 174 cells, control: 1.91±0.13; *GluA4^HC−/−^*: 2.47±0.18, *p* = 0.018) and their firing fields were smaller (Figure 6K, control *n* = 407 fields, *GluA4^HC−/−^ n* = 429 fields, *p*<10*^−^* ^7^). We did not observe differences between pyramidal cells from *GluA4^HC−/−^* and control mice in regards to their remapping properties (Text S1). Global remapping between trials in the open field and the zigzag maze was observed in both genotypes (Figure S8).

### Reduced Correlation between Theta Phase and Position in *GluA4^HC−/−^* Mice

**Figure 7:**
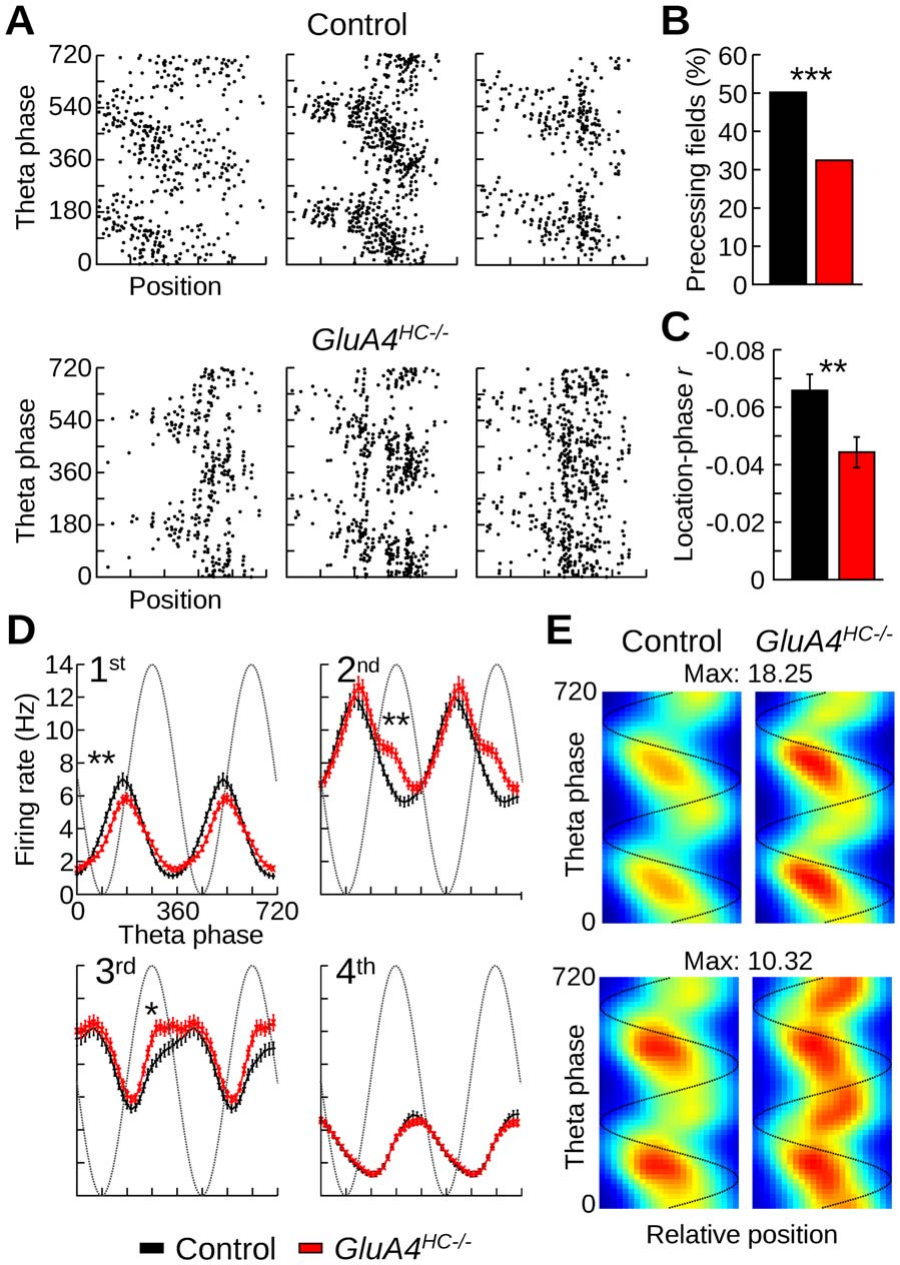
Theta phase precession in control and *GluA4^HC−/−^* mice. (A) Theta firing phase versus distance traveled through the field for three place fields recorded in control and *GluA4^HC−/−^* mice in the zigzag maze. (B) Percentage of firing fields for which there was a negative correlation (*p*<0.05) between the theta phase of the spikes and position within the field. (C) Mean *r* value (± SEM) between theta phase and position for firing fields in control and *GluA4^HC−/−^* mice. (D) Mean firing rate of pyramidal cells at different theta phases in 4 segments of the firing fields. The first and last segments correspond to the beginning and end of the firing field, respectively. (E) Mean spatio-temporal map of the firing fields in control and *GluA4^HC−/−^* mice. The x-axis is the relative position of the mouse within the firing field. Top row: spatio-temporal map for fields with significant theta phase precession. Bottom row: spatio-temporal map for fields without significant theta phase precession. *: *p*<0.05, **: *p*<0.01, *** *p*<10*^−^* ^5^.

We found that pyramidal cells from *GluA4^HC−/−^* mice fired more at the peak and early descending phase of theta oscillations than pyramidal cells from control mice. We therefore assessed how this change would affect theta phase precession as the mouse ran into the firing fields of pyramidal cells. Theta phase precession was assessed in mice during the first daily trial in the zigzag maze. For each firing field, a circular-linear correlation was calculated between the theta phase of the spikes and the position of the mouse within the field. Examples of how the theta phase of spikes evolved as the mouse ran through the firing fields are shown in Figure 7A. Theta phase precession could be observed in several firing fields. However, the proportion of fields with significant theta phase precession was lower in *GluA4^HC−/−^* than in control mice (Figure 7B, control: 142 fields out of 283, *GluA4^HC−/−^* : 112 fields out of 345, *p* = 10*^−^* ^6^). Moreover, the distributions of *r* values between theta phase and position within the field were significantly different between genotypes (Figure 7C, control *n* = 283 fields, *GluA4^HC−/−^ n* = 345 fields, *p* = 0.0013).

We divided firing fields into 4 equal segments and calculated the firing rate of the cells at different theta phases (Figure 7D). The firing rate of pyramidal cells in both genotypes showed theta phase precession, but pyramidal cells of *GluA4^HC−/−^* mice tended to fire more than those of controls near the peak of theta oscillations. This difference was most clearly seen when the mouse was located it the middle segments of the firing fields. Differences in firing rates were tested at phase 90, 180, 270 and 360° for each of the 4 segments. In the first segments, the firing rate of pyramidal cells in *GluA4^HC−/−^* mice was lower than that of controls at phase 90° (control *n* = 283 fields, *GluA4^HC−/−^ n* = 345 fields, *p* = 0.009). In the second and third segments, the firing rate of pyramidal cells in *GluA4^HC−/−^* mice was significantly higher than that in control mice specifically at phase 270° (second segment: *p* = 0.00017, third segment: *p* = 0.01). There was no significant difference between the firing rate of pyramidal cells in the two genotypes in the fourth segment of the place field.

A spatio-temporal map was calculated for each field. Fields with or without significant theta phase precession were averaged (Figure 7E). Firing fields that did not show significant theta phase precession in *GluA4^HC−/−^* mice had higher firing rate at the peak of theta oscillations. Similar findings were observed during the second daily trial in the zigzag maze (data not shown).

## Discussion

In this study we selectively reduced AMPA receptor-mediated excitatory currents onto a subset of GABAergic interneurons by ablating GluA4 in the hippocampus. Because the genetic manipulation was limited to the hippocampus, any extra-hippocampal alteration can be excluded from possible causes for the observed phenotype. It was previously shown that of the GluA4-expressing GABAergic interneurons in the CA1 region, approximately 80% express parvalbumin. Accordingly, in mice with global GluA4 ablation, AMPA receptor-mediated currents were affected mainly in fast-spiking parvalbumin-expressing interneurons, and there was a small AMPA receptor-mediated current reduction in a subpopulation of calbindin-expressing interneurons [19]. The preferential GluA4 expression in fast-spiking parvalbumin-expressing cells suggests that this cell population plays a major role in the *GluA4^HC−/−^* phenotype, but we cannot exclude a participation of other interneurons.

Hippocampal specific ablation of GluA4 resulted in several alterations of SWRs recorded in the CA1 pyramidal cell layer. SWRs recorded *in vivo* are preceded by a marked increase in CA3 pyramidal cell activity [38]. This activity burst leads to the depolarization of CA1 pyramidal cells and interneurons, which triggers ripples in the pyramidal cell layer of the CA1 region. The mechanisms responsible for the ripple generation (the rhythm generator) are still not fully understood but could involve electrical coupling between the axons of CA1 pyramidal cells [39], [40] and/or interactions between CA1 pyramidal cells and inhibitory interneurons [12], [13]. In *GluA4^HC−/−^* mice, ripples had a larger amplitude. The current responsible for ripples recorded in the local field potentials are thought to involve synchronized somatic IPSPs interrupted by the synchronous discharge of CA1 pyramidal cells [13], [15]. Interneurons in *GluA4^HC−/−^* mice had normal firing rate, preferred ripple phase and modulation of firing rate on each cycle. In contrast, CA1 pyramidal cells in mutant mice were more active during ripples. A larger summation of spikes near the trough of the ripples in *GluA4^HC−/−^* mice could have contributed to the increased ripple amplitude. Moreover, the higher firing rate of pyramidal cells in mutant mice suggests that their membrane potential was on average more depolarized. Therefore, a rhythmic release of GABA onto these pyramidal cells could generate stronger inhibitory currents and contribute to the increased amplitude of ripples observed in *GluA4^HC−/−^* mice [13]. Noteworthy, a clear relationship between the firing rate of pyramidal cells and the magnitude of SWRs was also observed in rats [41].

There could be several alterations in *GluA4^HC−/−^* mice responsible for the increased firing rate of CA1 pyramidal cells during SWRs. CA1 pyramidal cells receive their main excitatory inputs from the entorhinal cortex and CA3 pyramidal cells. Since our molecular manipulation did not affect the entorhinal cortex, the most likely extra-CA1 mechanisms lie within the CA3 region. Reduced feedback inhibition within the CA3 network could lead to a larger burst of activity in CA3 pyramidal cells during SWRs. In addition, the increased firing of CA1 pyramidal cells during SWRs could be caused by reduced feedforward and/or feedback inhibition within the CA1 network. It should be noted that we observed a decrease in the overall frequency of occurrence of SWRs in *GluA4^HC−/−^* mice, indicating that some of the mechanisms responsible for SWRs initiation are located within the hippocampus [42].

It was previously shown that an ablation of the GluA1 subunit of the AMPA receptor in parvalbumin-expressing interneurons (*GluA1^PV−/−^* mice) resulted in increased ripple amplitude in the CA1 region [18]. However, the mechanisms behind the increased ripple magnitude in *GluA4^HC−/−^* mice differed from those in *GluA1^PV−/−^* mice. In the latter mouse model, larger ripple amplitude was associated with stronger rhythmic modulation of interneuron and pyramidal cell firing. No change in the firing rate of pyramidal cells was observed. These differences in the putative underlying mechanisms responsible for the increased ripple amplitude in *GluA4^HC−/−^* and *GluA1^PV−/−^* mice could originate from different kinetics of the AMPA receptor-mediated current in interneurons [19], from a difference in the subsets of interneurons with reduced AMPA receptor-mediated currents or from other extra-hippocampal alterations in *GluA1^PV−/−^* mice.

SWRs are thought to contribute to the consolidation of new memory traces [1]. In support of this hypothesis, exploratory activity patterns of hippocampal pyramidal cells reoccur during SWRs [8], [43]–[45]. In this context, the increased firing probability of CA1 pyramidal cells during SWRs in *GluA4^HC−/−^* mice suggests that more pyramidal cells take part in the reactivation of memory traces in mutant mice. Thus, the recruitment of GluA4-expressing interneurons could contribute to the selection of pyramidal cells active during reactivation episodes.

The spatial selectivity of pyramidal cells in *GluA4^HC−/−^* mice was similar to that of control mice. The spatial information content of spikes and spatial sparsity in *GluA4^HC−/−^* mice were not significantly different from those of controls. Although we observed modest changes of place field size in two environments, these changes were not indicative of a general loss of spatial selectivity in pyramidal cells. Several computational models have proposed mechanisms by which the periodic activity of grid cells could be transformed into the sparse spatial representation typical of place cells [5], [22]–[28]. The sparse hippocampal code often depends on competition between hippocampal pyramidal cells which is implemented via feedforward or feedback inhibitory connections. In one model [23], the influence of the strength of feedback inhibition in the hippocampus on the sparsity of hippocampal place cells was directly tested; there was a positive relationship between the strength of inhibition and sparsity of firing. Although AMPA receptor-mediated currents onto fast-spiking interneurons were reduced in *GluA4^HC−/−^* mice, we did not observe a significant degradation of spatial selectivity. This suggests that the proposed competition between hippocampal pyramidal cells does not depend critically on the GluA4 component of AMPA receptor-mediated currents in hippocampal interneurons. Instead, remaining excitatory currents onto interneurons expressing GluA4 could be sufficient to achieve normal competition levels between pyramidal cells. It is also possible that this competition is implemented by dendrite-targeting interneurons that do not express GluA4 at a high level [19], [46].

We found that a reduction in AMPA receptor-mediated currents onto hippocampal interneurons led to a reduction in the proportion of place fields with significant theta phase precession. The mean correlation between theta phase of spikes and position was also weaker in *GluA4^HC−/−^* mice. Different mechanisms have been put forward to explain theta phase precession in the hippocampus. The simplest scenario is that theta phase precession in the CA1 region is passively inherited from phase precessing excitatory inputs from grid cells, either monosynaptically or indirectly via CA3 pyramidal cells [47]. Alternatively, theta phase precession in the hippocampus could originate from interactions between dendritic depolarization of CA1 pyramidal coupled with rhythmic perisomatic shunting inhibition provided by fast-spiking interneurons [31], [48]–[51]. The alteration of theta phase precession after selective reduction of AMPA receptor-mediated currents onto hippocampal inhibitory interneurons suggests that local inhibition plays a significant role in theta phase precession.

GluA4 ablation in *GluA4^HC−/−^* mice resulted in a severe spatial working memory deficit. In fact, this deficit was more pronounced than that in mice with global GluA4 ablation [19]. The behavioral differences that resulted from hippocampus-specific and global GluA4 ablation possibly reflect developmental compensatory mechanisms in mice with global GluA4 ablation. The behavioral phenotype observed in *GluA4^HC−/−^* mice is in line with that reported by Murray and colleagues [21]. These authors showed that blocking synaptic release from CA1 parvalbumin-expressing interneurons resulted in spatial working memory deficits, but left spatial reference memory intact.

In *GluA4^HC−/−^* mice, the spatial selectivity of hippocampal cells was mostly preserved but spatial working memory performance was severely impaired. Although a reduction of spatial selectivity of place cells has been linked to impaired spatial working memory performance [52], [53], it seems not very likely that a change in the spatial selectivity of hippocampal place cells in *GluA4^HC−/−^* mice was responsible for their impaired spatial working memory. It has been proposed before that the precise temporal coordination of pyramidal cell activity during theta oscillations is critical for spatial working memory performance [54]. The results presented in our study are consistent with this hypothesis.

## Methods

### Ethics Statement

All experiments were carried out in accordance with the European Committees Directive (86/609/EEC) and were approved by the Governmental Supervisory Panel on Animal Experiments of Baden Württemberg at Karlsruhe (35-9185.81/G-113/10 and 35-9185.81/G-91/11).

### Virus Production and Injection into Dorsal Hippocampus

The recombinant adeno-associated virus expressing Cre recombinase (AAV-Cre) contained the CMV immediate early enhancer/chicken β-actin hybrid promoter, the nuclear localization signal of the simian virus 40 large T antigen, the Cre recombinase coding region, the woodchuck posttranscriptional regulatory element (WPRE), and the bovine growth hormone poly(A) [55]. AAV chimeric vectors (virions containing a 1∶1 ratio of AAV1 and AAV2 capsid proteins with AAV2 inverted terminal repeats) were generated as previously described [56]. HEK293 cells were transfected using standard calcium phosphate transfection method with the AAV cis plasmid, the AAV1 and AAV2 helper plasmids, and the adenovirus helper plasmid. Sixty hours after transfection, cells were harvested and the vector was purified using heparin affinity columns (Sigma, Taufkirchen, Germany). Genomic titers were determined using the ABI 7700 real time PCR cycler (Applied Biosystems, Carlsbad, CA) with primers designed to WPRE [57]. AAV-Tomato was constructed and produced similarly to the AAV-Cre.

Virus injections were performed in 8–9 weeks old mice anesthetized with isoflurane. AAV-Cre (titer 2×10^13^ viral genomes/ml) was injected into the dorsal hippocampus (2.4 mm posterior and 2 mm lateral from bregma, 1.5 mm below brain surface, 1 µl over 2–3 min in each hemisphere). Eight *ROSA26* reporter mice [58] were injected to assess the reliability of the Cre-mediated recombination in the hippocampus. Wildtype and homozygous *GluA4^2lox^* littermates with a C57/Bl6-N background [19] were injected with AAV-Cre or AAV-Tomato. In behavioral and *in vivo* electrophysiological experiments, mice were analyzed 7–8 weeks after virus injection. In *in vitro* electrophysiological experiments, 6 week old mice were injected and recordings were performed 4–6 weeks later.

### Western Blot Analysis

Western blots were performed to assess GluA4, GluA1 and GluN1 expression in the hippocampus, the entorhinal cortex, the dorsolateral nuclei of the thalamus and the visual cortex. To compare expression in the dorsal and ventral hippocampus, single hippocampi were dissected into dorsal and ventral segments. Three dorsal or ventral hippocampal segments of the same genotype were pooled to obtain sufficient protein for reliable quantification. For details see text S1.

### *In vitro* Electrophysiology

Dorsal hippocampal slices were prepared from control and *GluA4^HC−/−^* mice. Mice were deeply anesthetized with isoflurane and killed by decapitation. The brain was removed and submerged in ice-cold high-sucrose ACSF containing (in mM): 212 sucrose, 3 KCl, 1.25 NaH_2_PO_4_, 26 NaHCO_3_, 7 MgCl_2_, 0.5 CaCl_2_, 10 glucose continuously bubbled with 95% O_2_ and 5% CO_2_ (pH 7.3). Transverse slices (250 µm) were cut in high-sucrose ACSF using a vibratome (HR2; Sigmann Elektronik, Germany). Slices were kept at 32°C for 30 min in physiological ACSF containing (in mM), 125 NaCl, 25 NaHCO_3_, 1.25 NaH_2_PO_4_, 2.5 KCl, 2 CaCl_2_, 1 MgCl_2_, 25 glucose, continuously bubbled with 95% O_2_ and 5% CO_2_ (pH 7.3). Recording pipettes made of borosilicate glass (resistance 4–6 MΩ) were filled with intracellular solution containing (in mM): 120 Cs-gluconate, 10 CsCl, 8 NaCl, 10 HEPES, 10 phosphocreatine-Na, 0.3 Na_3_GTP, 2 MgATP, 0.2 EGTA (pH 7.3, adjusted with NaOH). Liquid junction potentials were not corrected. Whole-cell voltage clamp recordings were performed in neurons located at the border between stratum oriens and stratum pyramidale (fast-spiking cells), or in stratum pyramidale (pyramidal cells). Cells were visually identified by infrared differential-contrast videomicroscopy. Immediately after rupturing the patch, the resting membrane potential was measured in current-clamp and the firing pattern was examined. Fast-spiking cells were identified by their characteristic high-frequency firing pattern (maximal firing frequency >150 Hz) in response to 1s-long current injections. Pyramidal cells were identified by the shape of their soma and their adapting firing pattern. Extracellular stimulation was performed at 0.1 Hz with a stimulation pipette filled with physiological ACSF. The stimulation pipette was positioned in the stratum radiatum of the CA1 region for the recordings of pyramidal cells and in the stratum oriens of the CA1 region, towards the CA3 region, for the recordings of fast-spiking cells. All recordings were performed in the presence of gabazine 10 µM (SR 95531, Biotrend, Germany) to block GABA_A_ receptors. For AMPA/NMDA ratios, the AMPA component was measured at the peak of the EPSC recorded at -70 mV. The NMDA component was measured at +70 mV, 25 ms after stimulation. To avoid maintaining the cell at +70 mV, the recorded cell was held at +40 mV, and briefly depolarized for 1 s at +70 mV before stimulation. Decay times were calculated using a monoexponential fit (IGOR Pro, WaveMetrics).

### Assessment of Spatial Memory

Spatial memory tests were conducted in age-matched male *GluA4^HC−/−^* and control mice. As the performance of *GluA4^2lox^* injected with AAV-Tomato (9 mice) and wildtype mice injected with AAV-Cre (7 mice) was indistinguishable, the data were pooled and compared to that of 13 *GluA4^HC−/−^* mice. Mice were maintained on a restricted feeding schedule to maintain them at 85% of their free-feeding weight. They were first tested on a spatial reference memory test followed by a spatial working memory test.

Spatial reference memory was tested on an elevated Y-maze, a behavioral test sensitive to hippocampal lesions in mice [35]. The maze was made of black painted wood. It had a central polygonal area with a diameter of 14 cm to which three arms were attached (50×9 cm, surrounded by a 0.5 cm high ridge). A plastic food well was located 5 cm away from the distal end of each arm. The maze was surrounded by prominent distal cues and was elevated 80 cm above the ground. The entire maze could be rotated to prevent the use of intra-maze cues. Mice were familiarized to the maze until they ran freely on the maze. A target arm (defined according to its given spatial location relative to the room cues) was designated for each mouse to receive 0.1 ml of sweetened condensed milk as a reward. Target arms were counterbalanced with respect to the genotype of the mice. The start arm for each trial was determined by a pseudorandom sequence with equal numbers of starts from each arm in any given session, and no more than three consecutive starts from the same arm. The mouse was placed at the distal end of a start arm, and was allowed to run freely until it found its reward. A correct choice was recorded when the mouse entered the target arm before entering any other arm.

Spatial working memory was assessed with a rewarded alternation task on an elevated T-maze. Mice with hippocampal lesions are profoundly impaired on this task [35]. The mice were habituated to the maze over several days before spatial non-matching-to-place testing. During the test, each trial consisted of a sample run and a choice run. On the sample run, the mouse was forced either left or right by the presence of a wooden block, according to a pseudorandom sequence (with equal numbers of left and right turns per session, and not more than two consecutive trials with the same direction). A reward (0.1 ml of sweetened condensed milk) was available in the food well at the end of the arm. The block was then removed, and the mouse placed, facing the experimenter, at the end of the start arm. The delay interval between the sample run and the choice run was 10–15 s. The mouse was rewarded for choosing the previously unvisited arm (i.e. for alternating). Mice were run one trial at a time with an inter-trial interval of approximately 10 min. Mice received 4 trials per day for 10 days.

*GluA4^HC−/−^* and control mice were also tested on a version of the radial arm maze assessing both reference and working memory [59]. The maze was elevated 80 cm above the floor in a well-lit room containing various extra-maze cues (e. g. laboratory equipment, stools, table and posters). Each of the 8 arms (30×6 cm) extended radially from a circular platform (19 cm diameter). A food well was located at the end of each arm. At the entrance of each maze arm there was a gray Perspex door that could be operated from a distance by the experimenter.

The mice were first habituated to drinking sweetened condensed milk on two arms of an elevated Y-maze in a different room (i.e. not the testing room). Once all mice ran freely on the Y-maze and readily consumed the milk reward, testing on the radial arm maze began. The mice were trained on a radial arm maze task in which the same four arms were always baited and the milk rewards were not replaced within a trial. The four baited arms were allocated such that two of the arms were adjacent, and the other two arms were 90° apart from the adjacent arms (e.g. arms 1,3,6,8). The baited arms were kept constant throughout the training. Different combinations of arms were used across mice and they were counterbalanced across groups.

At the start of a trial, a mouse was placed individually on the central platform. Mice were allowed to explore freely and consume all milk rewards available. During the acquisition phase, the doors prevented mice from re-entering an arm they had already visited on that trial. All doors were closed each time the mouse returned to the central platform where the mouse was confined for 10 s until the next choice. Once an arm had been visited, its door remained closed for subsequent choices to prevent working memory errors. Spatial reference memory errors were defined as entries into arms that were never baited (maximum of 4 errors per trial). The maze was rotated periodically to prevent the mice from using intra-maze cues to solve the task. Mice received 60 trials and the data were analyzed in blocks of 4 trials. By this stage all animals had acquired the spatial reference component of the task and made few errors.

The spatial working memory component of the task was then introduced. The mice received further 28 trials (with an inter-choice interval of 10 s) in which the same 4 out of 8 arms were baited, but now they were no longer prevented from re-entering a previously chosen arm. The doors were solely used to confine the mice on the central platform between choices. Spatial working memory errors were scored when a mouse entered an arm that had been already visited on that trial. Spatial reference memory errors were scored as before.

### Tetrode Implantation and Electrophysiological Recording During Behavior

Nine control and 12 *GluA4^HC−/−^* mice were anesthetized with isoflurane and implanted with 4 to 8 independently movable tetrodes targeted at the CA1 region. Tetrodes were constructed from 12-µm-diameter tungsten wires (H-Formvar insulation with Butyral bond coat; California Fine Wire, Grover Beach, CA). During surgery, the dura mater above the hippocampus (2.2 mm posterior and 1.8 mm lateral to bregma) was removed and the tetrodes were inserted 0.5 mm deep into the cortex. Two stainless steel screws were implanted into the bone above the cerebellum and served as reference and ground signals.

After a recovery period of one week, the tetrodes were lowered into the CA1 region over approximately 2 weeks. Tetrodes were considered to be located in the CA1 pyramidal cell layer if ripples (125–200 Hz) were observed during immobility and sleep. Only tetrodes located in the CA1 pyramidal cell layer were analyzed. Following each recording session, the tetrodes were moved down 25–50 µm. Electrode placement was subsequently confirmed by histological analysis.

The brain signal was passed through a channel unity-gain preamplifier headstage (TLC2274C, Texas Instruments) before being amplified (x 600) and sampled at 24 kHz (16 bits/sample, DacqUSB, Axona Ltd.). Three light-emitting diodes were attached to the headstage to track the position of the animal during recording. The video signal from a camera mounted on the ceiling was analyzed by custom-made position tracking software (25 Hz, resolution 6 pixels/cm).

### Behavioral Paradigm for Electrophysiological Recording

Following the recovery period, mice were maintained at 85–90% of their free-feeding weight and trained to forage for food in two different environments: an open field (70×70×30 cm high) and a zigzag maze. The zigzag maze consisted of five 7-cm wide, adjacent alleys. Each alley was 65 cm long and walls were 20 cm high. The walls separating the alleys were 58 cm long. Both apparatuses were made of wood painted dark gray. Open-field exploration was performed to measure the spatial selectivity of place cells in a conventional, 2-dimensional environment. We used the zigzag maze to quantify theta phase precession.

During open-field training, each mouse received two 10-min daily trials during which food crumbs were delivered every 20–40 sec. This was repeated for 5 days. Thereafter, the mouse was connected to the recording system before being placed in the open field for training. Training continued until the mouse explored readily the entire open field.

For the familiarization to the zigzag maze, a mouse was first placed on the maze with food available at random locations for two 10-min daily trials. On subsequent days, the food rewards were moved progressively away from the center of the maze until they were only available at the two ends of the maze. After approximately 10 days, the recording cable was attached to the mouse prior to the training sessions. Training continued until the mouse ran from one end to the other without much hesitation.

Each recording session consisted of nine 20-min trials, starting with a rest trial and alternating between foraging and rest trials. The foraging trials took place in the open field and the zigzag maze, following a ABAB protocol. The order of presentation of the open field and the zigzag maze was counterbalanced across recording sessions. During rest trials, the mouse was placed in a rest box (23×25×30 cm high) for 20 min and did not receive food reward.

### Analysis of Electrophysiological Data

Cell activity and oscillatory field potential patterns were analyzed off-line with C/C++ programs and shell scripts on computers running a GNU/Linux operating system. Spikes were extracted from the bandpass filtered (0.8–5 kHz) signal and their features were obtained using principal components analysis [15]. Spikes were grouped into putative individual neurons with automatic clustering software (http://klustakwik.sourceforge.net/), before being manually refined. Only clusters that were stable for the duration of a recording session and with a clear refractory period in their spike-time autocorrelation were processed further.

Pyramidal cells and interneurons were sorted based on the first moment of their spike-time autocorrelation (25 ms time window), their firing rate during the entire recording session and their mean spike duration measured at 25% of the maximum spike amplitude. We made the assumption that most of the highly spatially selective neurons in the CA1 pyramidal cell layer are pyramidal cells and selected neurons from control mice with a spatial information score >0.5 during open-field exploration. These neurons were used to build a template of pyramidal cells. The Mahalanobis distance of every recorded neurons from that template was calculated in 3-dimensional space. Neurons with a Mahalanobis distance <20 were classified as pyramidal cells whereas neurons with a distance >40 were classified as interneurons. Neurons with a distance between 20 and 40 were left unclassified and were not analyzed further. The quality of cluster isolation for pyramidal cells was quantified by calculating the isolation distance for each cluster [60], [61].

#### Sharp wave/ripples detection and analysis

SWRs were detected on tetrodes located in the CA1 pyramidal cell layer after applying a band-pass filter (125–250 Hz) to the raw signal. A channel from a tetrode located outside the CA1 pyramidal cell layer served as a reference channel to eliminate contamination of the detection by muscle artifacts. The power (root mean square) was calculated in 20 ms windows (10 ms between windows) and a window with a power larger than 7 SD above the mean was considered as part of a SWR epoch. The epoch extended in both directions until a window with a power below 2.5 SD above the mean was encountered. Individual cycles of the ripples were detected within these epochs from the band-pass filtered signal. Time-frequency power representations of SWRs were computed with a continuous wavelet transform method using Morlet wavelets with a SD of one period. Each wavelet was normalized so that they all had the same total energy. The raw signal and the wavelet at one frequency were convoluted using a fast Fourier transform. For each bin of the time-frequency map, the absolute value of the convolution is presented in the density plots. This time-frequency representation was calculated for each SWR epoch and averaged across all epochs for a given recording session. Power spectra of SWR epochs were computed using a multitaper method [62]. The power spectra from all tetrodes of one recording session were averaged, and a mean power spectrum from different recordings sessions was calculated for each mouse.

#### Theta detection and theta modulation of cell activity

Theta oscillations were detected on each tetrode located in the CA1 pyramidal cell layer. The signal was bandpass filtered at delta (2–4 Hz) and theta (6–10) frequency and the power of the filtered signal (root mean square) was calculated for each 500 ms window. Theta epochs were defined as windows with a theta/delta power ratio >2. Within these epochs, the raw signal was filtered at 5–14 Hz, and the positive-to-negative zero-crossings were detected and assigned to phase 0°. The theta firing phase of spikes was linearly interpolated between these points. Power spectra of theta epochs were computed using a multitaper method [62] and were calculated separately for periods when the mouse was running at different speed during foraging trials. The power spectra of all tetrodes from one recording session were averaged, and a mean power spectrum from different recordings sessions was calculated for each mouse.

The modulation of cell activity by theta oscillations was estimated for each neuron by calculating a mean vector length from the theta phase of its spikes [63]. The preferred phase of a neuron was defined as the circular mean of the spikes. The intrinsic theta frequency of neurons was estimated from the spike-time autocorrelation during theta epochs (time window from −200 to 200 ms, 2 ms bins). The time point associated with the maximum frequency between 70 and 180 ms (or 14.29 and 5.56 Hz) was considered the theta firing frequency of the cell.

We also analyzed in-field firing phase of neurons after dividing the firing fields into 4 segments of equal length on the position axis. Spikes were assigned to one of the segments depending on the position of the mouse when the spikes occurred. Theta phase precession was quantified for each place field by calculating a circular-linear correlation between the theta phase of the spikes and the location of the mouse within the field.

#### Spatial properties of neurons during open-field exploration

The data from the 2 foraging trials in the open field were analyzed separately and pyramidal cells firing less than 300 spikes during a trial were not considered. Only periods in which the mouse ran faster than 3 cm/s were included in the analysis. Firing rate maps were constructed by dividing the recording environment into 2×2 cm bins. The time spent in every bin was calculated and the resulting occupancy map was smoothed with a Gaussian kernel function (kernel SD = 3 cm). The firing rate map of each cell was obtained by dividing the number of spikes emitted in a given bin by the time spent there. The firing rate maps were smoothed as described above. A place field consisted of at least one bin with a firing rate above 5 Hz and a minimum of 10 adjacent bins (40 cm^2^) with a firing rate above 20% of the highest firing rate in the field. Spatial information score and sparsity of firing served to quantify the spatial selectivity of pyramidal cells [7].

#### Spatial properties of neurons in the zigzag maze

Firing rate maps for trials on the zigzag maze were first constructed using the same method as for the open-field trials, but the runs towards each of the two ends of the maze (northbound and southbound runs) were treated separately. The 2-dimensional position data was also transformed into 1 dimension as described previously [52]. The linear firing rate maps were calculated using the same procedure and parameters as for the 2-dimensional firing rate maps (2 cm bins, smoothing kernel SD = 3 cm), with the exception that the data were unidimensional. Linear place fields were defined as an area of the maze containing a bin with a firing rate above 5 Hz, and extended in both directions until the firing rate dropped below 20% of the bin with the highest firing rate.

### Statistical Analysis

Differences between genotypes in Western blot and *in vitro* electrophysiological data were investigated with Student’s *t*-tests. Behavioral experiments were analyzed with ANOVAs. For *in vivo* recordings, Wilcoxon’s rank-sum tests were used unless otherwise specified. Distributions of circular data were compared with Watson’s test for homogeneity of circular data.

## Supporting Information

Figure S1 **Virus-mediated GluA4 deletion in** ***GluA4^HC−/−^*** **mice did not affect extra-hippocampal regions or hippocampal expression of other glutamate receptor subunits.** (A) Representative Western blot of GluA4 expression in brain areas surrounding the hippocampus. The thalamus samples included the dorsolateral thalamic nuclei. The cortical samples included the primary and secondary visual cortices. (B) Quantification of Western blot data (control *n* = 12 mice, *GluA4^HC−/−^ n* = 12 mice, mean ± SEM). Data are expressed as percentage of control levels. (C) Representative Western blot of glutamate receptor subunits GluA1, GluA2/3, GluA4 and GluN1 in the hippocampus of control and *GluA4^HC−/−^* mice. (D) Quantification of expression levels of glutamate receptor subunits (control *n* = 17 mice, *GluA4^HC−/−^ n* = 19 mice, mean ± SEM, ***: *p*<0.001). Data are expressed as percentage of control levels. Brain tissue was obtained from mice that underwent behavioral tests.
(TIFF)

Figure S2 **Virus-mediated GluA4 deletion in** ***GluA4^HC^*** ^*−****/****−*^ **mice did not affect hippocampal anatomy and parvalbumin expression.** (A) Immunofluorescence staining of coronal sections revealed no difference in overall hippocampal morphology two months after injection in control and *GluA4^HC−/−^* mice. Upper panels: Expression of the neuronal marker NeuN. Lower panels: Expression of the Ca^2+^-binding protein calbindin expressed in granule cells of the dentate gyrus, mossy fibers and hippocampal interneurons. (B) Immunostaining for parvalbumin was performed in 7 hemispheres from 5 control mice and 8 hemispheres from 5 *GluA4^HC−/−^* mice. There was no difference in the number of parvalbumin-positive cells between control and *GluA4^HC−/−^* mice in the indicated subfields (mean ± SEM, CA1, control: 93.9±4.2, *GluA4^HC−/−^*: 88.3±5.6, CA3, control: 199.1±10.0, *GluA4^HC−/−^*: 182.3±11.1, DG, control: 67.4±4.2, *GluA4^HC−/−^*: 57.1±5.5). Scale bar: 50 µm. Abbreviations: PV: parvalbumin, CB: calbindin, DG, dentate gyrus.
(TIFF)

Figure S3 **Physiological identification of pyramidal cells and interneurons in control and** ***GluA4^HC^*** ^*−****/****−*^ **mice.** (A) Three-dimensional representation of isolated clusters (putative neurons) from control mice. The three axes are the mean firing rate, the first moment of the 50-ms spike-time autocorrelation, and spike duration. Neurons from control mice with a spatial information score >0.5 during open-field exploration (red dots) were used to build a template of pyramidal cells (see methods section). (B) Mahalanobis distance of neurons from the pyramidal cell template. Most neurons were located within a short distance from the pyramidal cell template, with fewer neurons located further away. Neurons with a Mahalanobis distance <20 (log_e_(20) = 3.00) were classified as pyramidal cells whereas neurons with a distance >40 (log_e_(40) = 3.69) were classified as interneurons. (C and D) Three-dimensional representation of neurons in control and *GluA4^HC−/−^* mice. (E) Mean isolation distance of pyramidal cells from other clusters recorded on the same tetrode in control and *GluA4^HC−/−^* mice (mean ± SEM). (F) Mean waveform of pyramidal cells and interneurons recorded in control and *GluA4^HC−/−^* mice. Abbreviations: Int., interneurons; Pyr., pyramidal cells.
(TIFF)

Figure S4 **Time spend at different running speed by the mice during the recording trials.** The distributions are shown separately for the three different environments used in the *in vivo* recording experiments (A, B and C). The insets show the mean (±SEM) running speed in each environment.
(TIFF)

Figure S5 **Cell activity during SWRs.** (A) Firing rate of pyramidal cells centered on the ripple peak power of each SWR. Time 0 was aligned to the positive-to-negative zero crossing of the ripples. (B) Same as A but for interneurons. (C) Proportion of SWRs in which an interneuron fire from 1 to 15 spikes. There was no significant difference between the two groups (all *p* values >0.09). The probability of firing 0 spike was 0.241±0.038 and 0.281±0.049 for interneurons of control and *GluA4^HC−/−^* mice, respectively (*p* = 0.25).
(TIFF)

Figure S6 **Local field potentials and cell activity during theta oscillations.** (A) Mean theta peak frequency at different running speed intervals. (B) Mean power at the theta peak frequency at different speed intervals. (C) Mean spike-time autocorrelation for pyramidal cells during theta epochs. (D) Mean spike-time autocorrelation for interneurons during theta epochs.
(TIFF)

Figure S7 **Place field size.** (A) Mean place field size during the first daily trial in the open for control and *GluA4^HC−/−^* mice. The mean place field size was calculated separately for each mouse and the average was obtained from the score of each mouse. There was no significant difference between genotypes (control *n*: 8, *GluA4^HC−/−^ n*: 12, *p* = 0.27). (B) Same as A but for the second daily trial in the open field (*p* = 0.35).
(TIFF)

Figure S8 **Global remapping in** ***GluA4^HC^*** ^*−****/****−*^ **mice.** (A) Correlation between map similarity of pyramidal cell pairs during two exploratory trials in the same environment or two exploratory trials in different environments. (B) Correlation between instantaneous firing rate correlations of pyramidal cell pairs during two exploratory trials in the same environment or two exploratory trials in different environments.
(TIFF)

Table S1 **Active and passive properties of fast-spiking interneurons in control and GluA4HC** *^−^* **/** *^−^* **mice.**
(DOC)

Table S2 **Number of cells recorded during** ***in vivo*** **experiments in control and** ***GluA4^HC^*** ^*−****/****−*^ **mice.**
(DOC)

Text S1 **Supporting methods and results.**
(DOC)
