# Supplementary figures and images for: Selective Reduction of AMPA Currents onto Hippocampal Interneurons Impairs Network Oscillatory Activity
Source: PLoS One. 2012 Jun 4;7(6):e37318. doi: 10.1371/journal.pone.0037318 (PMC3366956; doi:10.1371/journal.pone.0037318)

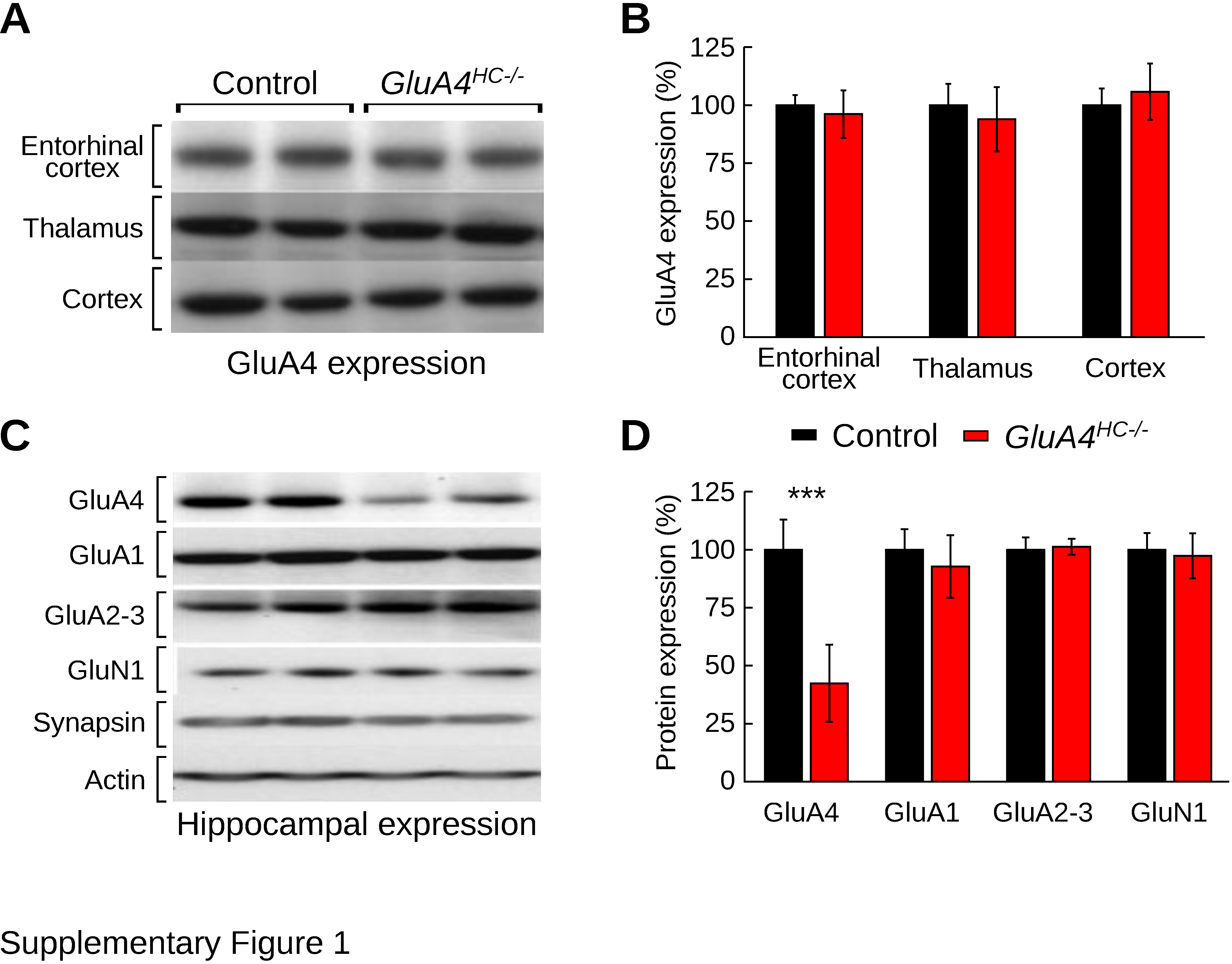

Supplement: Figure S1 — Virus-mediated GluA4 deletion in GluA4HC−/− mice did not affect extra-hippocampal regions or hippocampal expression of other glutamate receptor subunits. (A) Representative Western blot of GluA4 expression in brain areas surrounding the hippocampus. The thalamus samples included the dorsolateral thalamic nuclei. The cortical samples included the primary and secondary visual cortices. (B) Quantification of Western blot data (control n = 12 mice, GluA4HC−/− n = 12 mice, mean ± SEM). Data are expressed as percentage of control levels. (C) Representative Western blot of glutamate receptor subunits GluA1, GluA2/3, GluA4 and GluN1 in the hippocampus of control and GluA4HC−/− mice. (D) Quantification of expression levels of glutamate receptor subunits (control n = 17 mice, GluA4HC−/− n = 19 mice, mean ± SEM, ***: p<0.001). Data are expressed as percentage of control levels. Brain tissue was obtained from mice that underwent behavioral tests. (TIFF) [file pone.0037318.s001.tif]

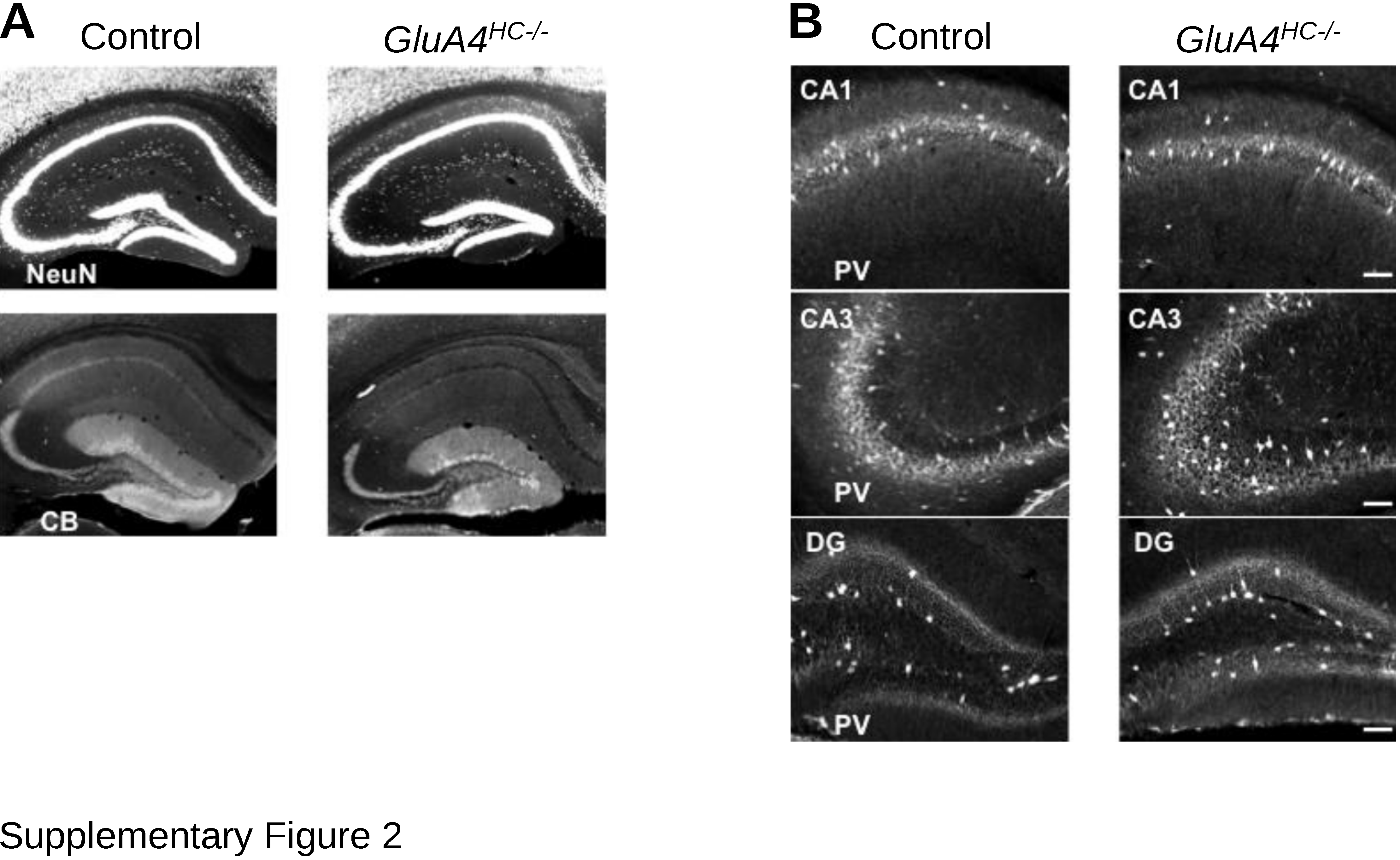

Supplement: Figure S2 — Virus-mediated GluA4 deletion in GluA4HC −/− mice did not affect hippocampal anatomy and parvalbumin expression. (A) Immunofluorescence staining of coronal sections revealed no difference in overall hippocampal morphology two months after injection in control and GluA4HC−/− mice. Upper panels: Expression of the neuronal marker NeuN. Lower panels: Expression of the Ca2+-binding protein calbindin expressed in granule cells of the dentate gyrus, mossy fibers and hippocampal interneurons. (B) Immunostaining for parvalbumin was performed in 7 hemispheres from 5 control mice and 8 hemispheres from 5 GluA4HC−/− mice. There was no difference in the number of parvalbumin-positive cells between control and GluA4HC−/− mice in the indicated subfields (mean ± SEM, CA1, control: 93.9±4.2, GluA4HC−/−: 88.3±5.6, CA3, control: 199.1±10.0, GluA4HC−/−: 182.3±11.1, DG, control: 67.4±4.2, GluA4HC−/−: 57.1±5.5). Scale bar: 50 µm. Abbreviations: PV: parvalbumin, CB: calbindin, DG, dentate gyrus. (TIFF) [file pone.0037318.s002.tif]

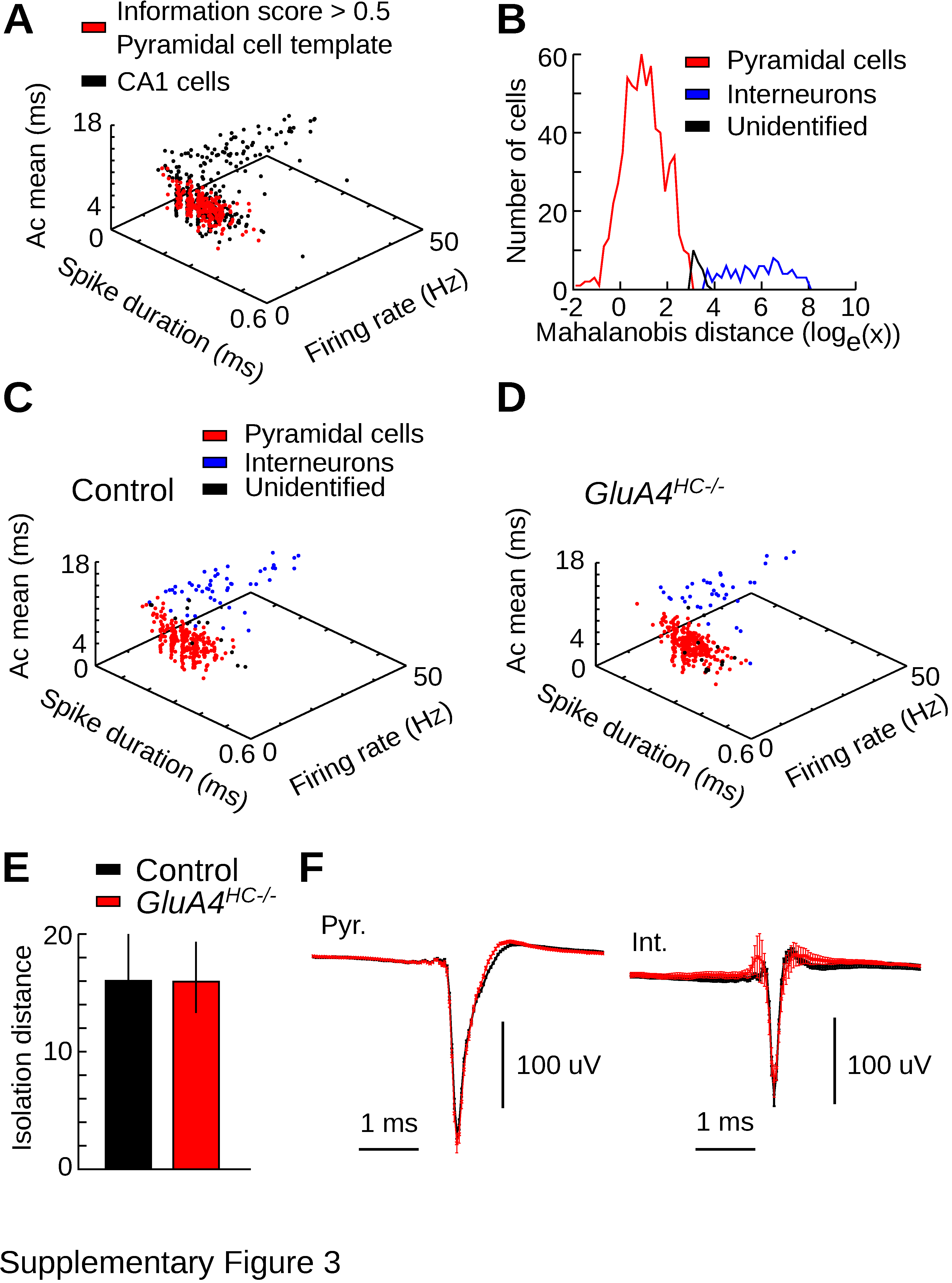

Supplement: Figure S3 — Physiological identification of pyramidal cells and interneurons in control and GluA4HC −/− mice. (A) Three-dimensional representation of isolated clusters (putative neurons) from control mice. The three axes are the mean firing rate, the first moment of the 50-ms spike-time autocorrelation, and spike duration. Neurons from control mice with a spatial information score >0.5 during open-field exploration (red dots) were used to build a template of pyramidal cells (see methods section). (B) Mahalanobis distance of neurons from the pyramidal cell template. Most neurons were located within a short distance from the pyramidal cell template, with fewer neurons located further away. Neurons with a Mahalanobis distance <20 (loge(20) = 3.00) were classified as pyramidal cells whereas neurons with a distance >40 (loge(40) = 3.69) were classified as interneurons. (C and D) Three-dimensional representation of neurons in control and GluA4HC−/− mice. (E) Mean isolation distance of pyramidal cells from other clusters recorded on the same tetrode in control and GluA4HC−/− mice (mean ± SEM). (F) Mean waveform of pyramidal cells and interneurons recorded in control and GluA4HC−/− mice. Abbreviations: Int., interneurons; Pyr., pyramidal cells. (TIFF) [file pone.0037318.s003.tif]

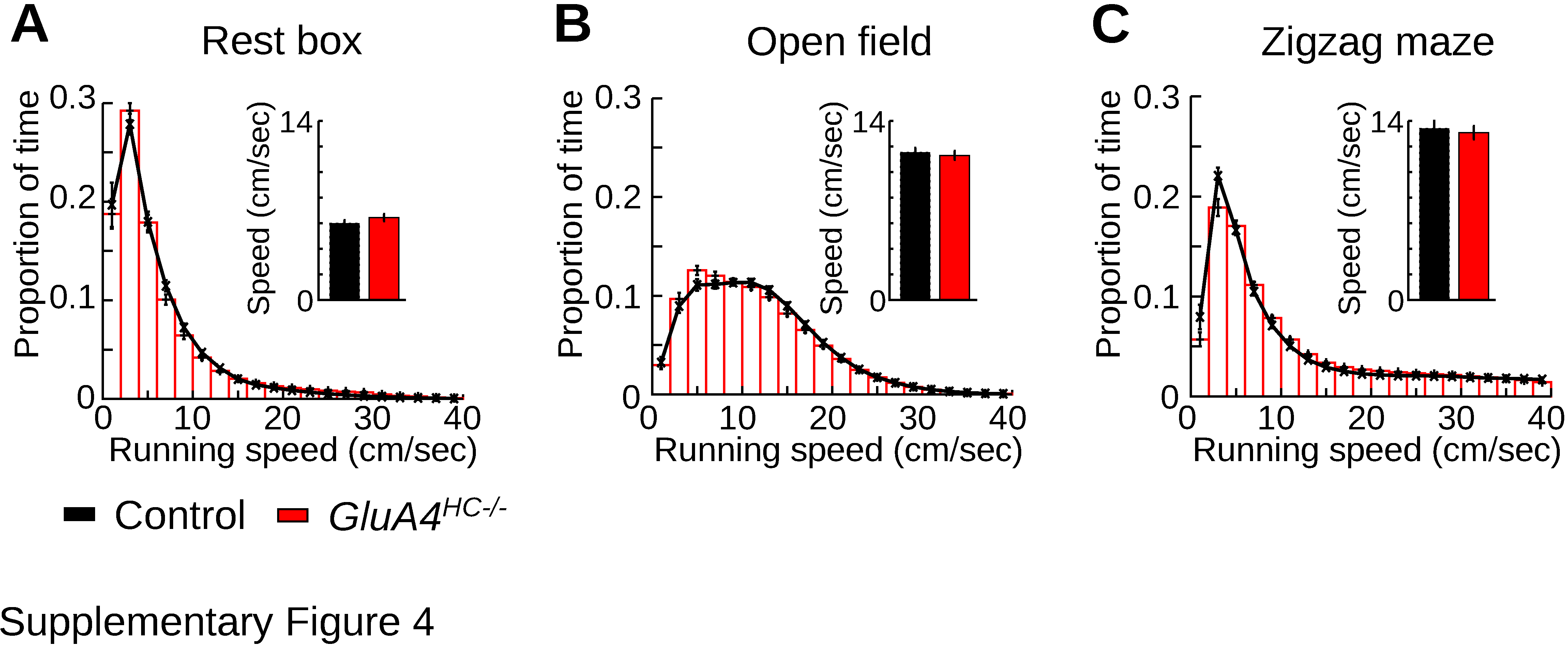

Supplement: Figure S4 — Time spend at different running speed by the mice during the recording trials. The distributions are shown separately for the three different environments used in the in vivo recording experiments (A, B and C). The insets show the mean (±SEM) running speed in each environment. (TIFF) [file pone.0037318.s004.tif]

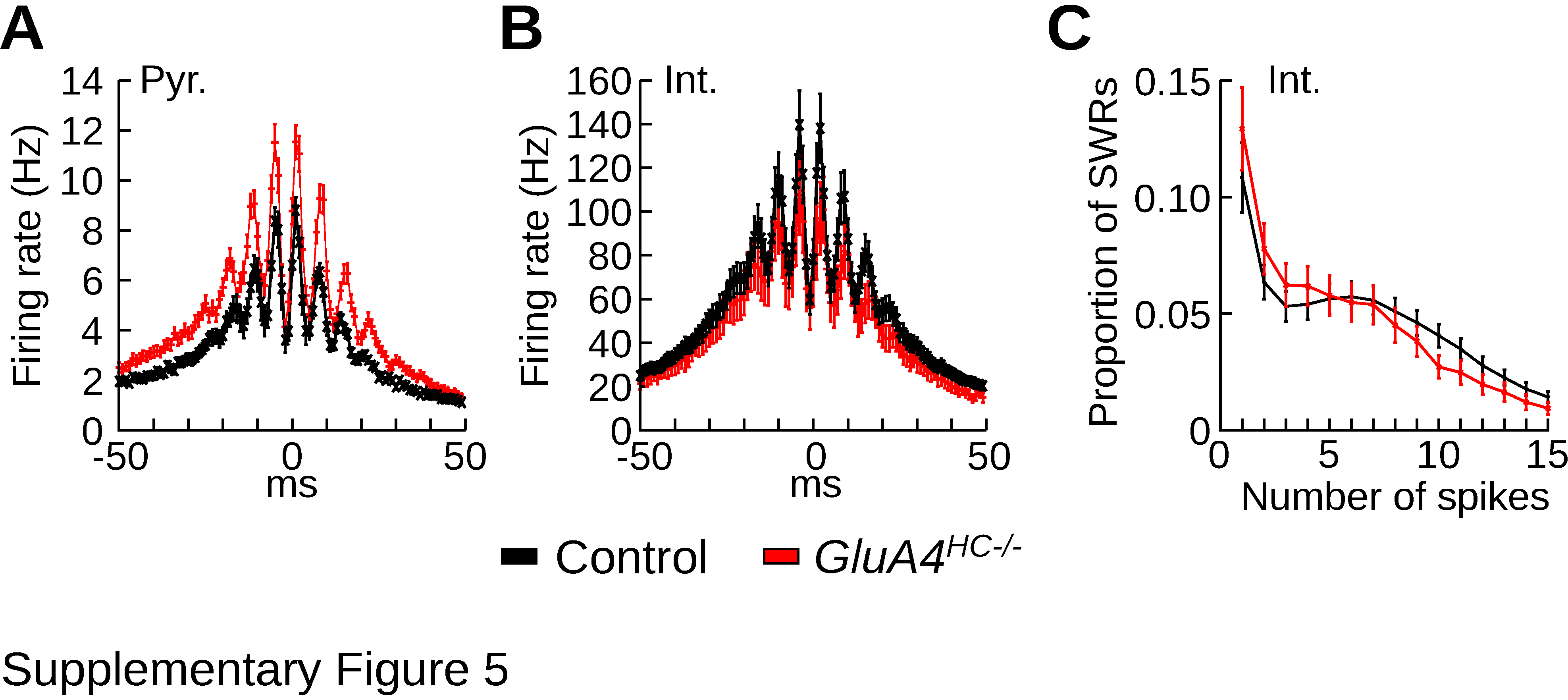

Supplement: Figure S5 — Cell activity during SWRs. (A) Firing rate of pyramidal cells centered on the ripple peak power of each SWR. Time 0 was aligned to the positive-to-negative zero crossing of the ripples. (B) Same as A but for interneurons. (C) Proportion of SWRs in which an interneuron fire from 1 to 15 spikes. There was no significant difference between the two groups (all p values >0.09). The probability of firing 0 spike was 0.241±0.038 and 0.281±0.049 for interneurons of control and GluA4HC−/− mice, respectively (p = 0.25). (TIFF) [file pone.0037318.s005.tif]

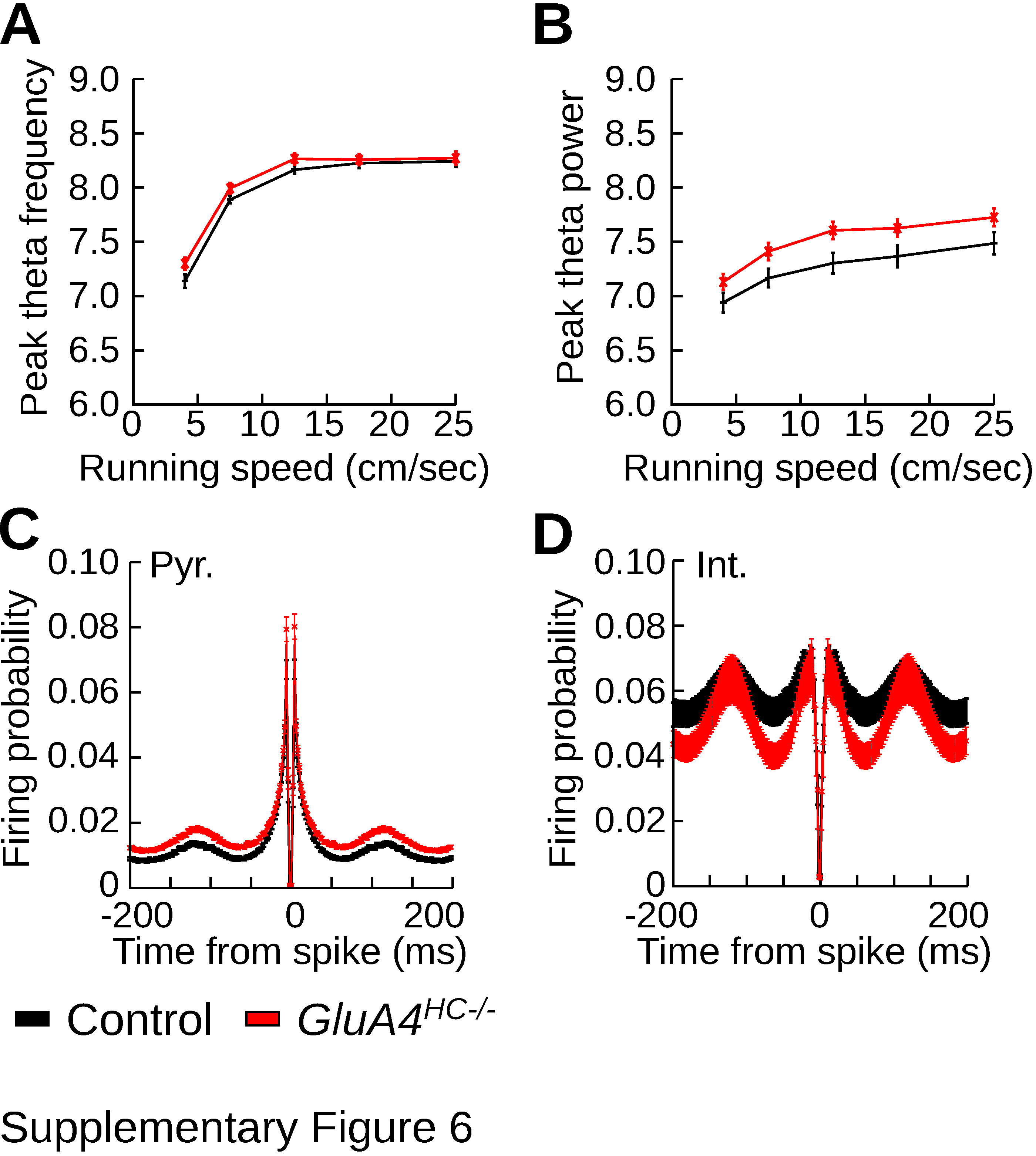

Supplement: Figure S6 — Local field potentials and cell activity during theta oscillations. (A) Mean theta peak frequency at different running speed intervals. (B) Mean power at the theta peak frequency at different speed intervals. (C) Mean spike-time autocorrelation for pyramidal cells during theta epochs. (D) Mean spike-time autocorrelation for interneurons during theta epochs. (TIFF) [file pone.0037318.s006.tif]

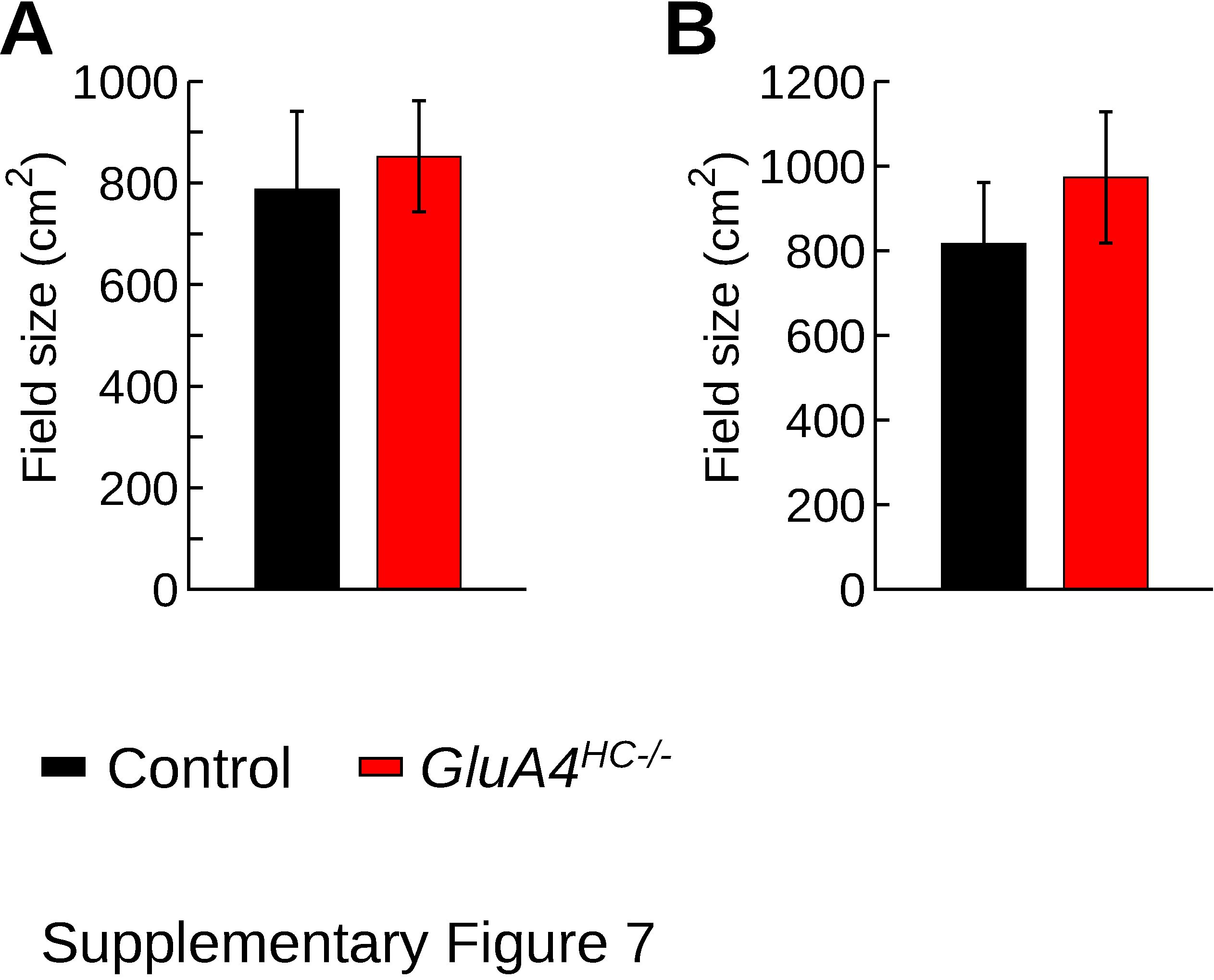

Supplement: Figure S7 — Place field size. (A) Mean place field size during the first daily trial in the open for control and GluA4HC−/− mice. The mean place field size was calculated separately for each mouse and the average was obtained from the score of each mouse. There was no significant difference between genotypes (control n: 8, GluA4HC−/− n: 12, p = 0.27). (B) Same as A but for the second daily trial in the open field (p = 0.35). (TIFF) [file pone.0037318.s007.tif]

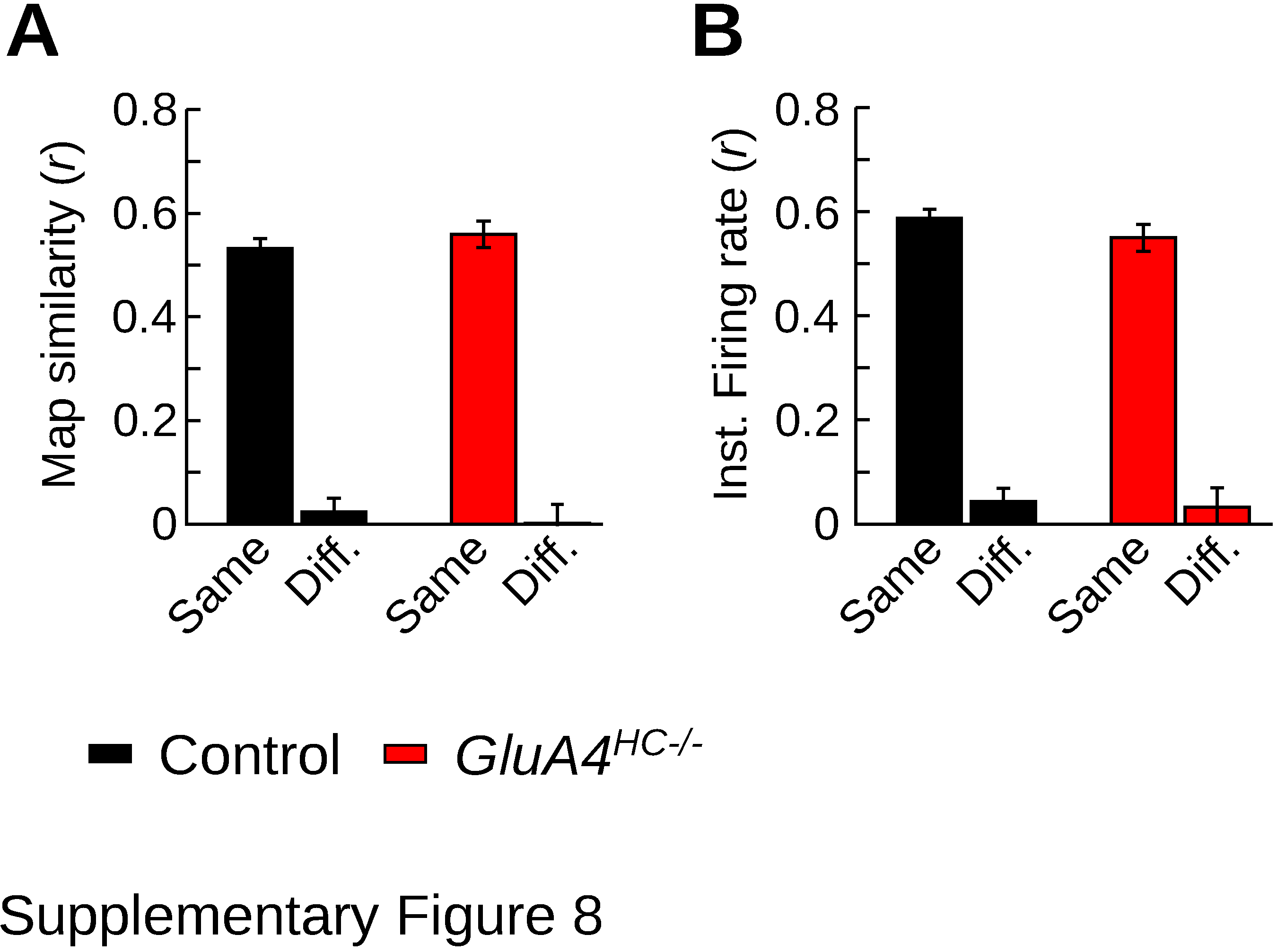

Supplement: Figure S8 — Global remapping in GluA4HC −/− mice. (A) Correlation between map similarity of pyramidal cell pairs during two exploratory trials in the same environment or two exploratory trials in different environments. (B) Correlation between instantaneous firing rate correlations of pyramidal cell pairs during two exploratory trials in the same environment or two exploratory trials in different environments. (TIFF) [file pone.0037318.s008.tif]
