# Supplementary material for: Selective Reduction of AMPA Currents onto Hippocampal Interneurons Impairs Network Oscillatory Activity
Source: PLoS One. 2012 Jun 4;7(6):e37318. doi: 10.1371/journal.pone.0037318 (PMC3366956; doi:10.1371/journal.pone.0037318)
Supplement: Table S1 — Active and passive properties of fast-spiking interneurons in control and GluA4HC − / − mice. (DOC) [file pone.0037318.s009.doc]

| **Fast-spiking interneurons** | Control | *GluA4^HC-/-^* | t-test |
| --- | --- | --- | --- |
| Active properties |  |  |  |
| Max firing rate (Hz) | 213 ± 12 | 186 ± 10 | *p* = 0.11 |
| Action pot. half-width (ms) | 0.75 ± 0.07 | 0.83 ± 0.06 | *p* = 0.48 |
| Passive properties |  |  |  |
| Membrane time constant (ms) | 14.7 ± 1.6 | 16.4 ± 1.9 | *p* = 0.53 |
| Input resistance (MΩ) | 132 ± 8.7 | 155 ± 16 | *p* = 0.24 |
| Resting membrane potential (mV) | -69.3 ± 1.3 | -73.0 ± 2.1 | *p* = 0.17 |
| Number of cells | 17 | 14 |  |
