# Supplementary material for: Selective Reduction of AMPA Currents onto Hippocampal Interneurons Impairs Network Oscillatory Activity
Source: PLoS One. 2012 Jun 4;7(6):e37318. doi: 10.1371/journal.pone.0037318 (PMC3366956; doi:10.1371/journal.pone.0037318)
Supplement: Table S2 — Number of cells recorded during in vivo experiments in control and GluA4HC −/− mice. (DOC) [file pone.0037318.s010.doc]

| **Control mice** | **Pyramidal cells** | **Interneurons** | **Unclassified cells** |
| --- | --- | --- | --- |
| ka1048 | 24 | 6 | 0 |
| ka1070 | 13 | 4 | 0 |
| ka1872 | 176 | 19 | 7 |
| ka195 | 60 | 21 | 3 |
| ka2396 | 39 | 4 | 2 |
| ka2413 | 30 | 9 | 0 |
| ka2536 | 13 | 2 | 1 |
| ka968 | 0 | 0 | 0 |
| ka981 | 7 | 0 | 0 |
| Total: 440 | 362 | 65 | 13 |
| ***GluA4^HC-/-^* mice** | **Pyramidal cells** | **Interneurons** | **Unclassified cells** |
| ka1000 | 35 | 2 | 0 |
| ka1047 | 1 | 0 | 0 |
| ka1293 | 6 | 0 | 0 |
| ka1326 | 41 | 3 | 0 |
| ka1440 | 26 | 9 | 1 |
| ka1889 | 13 | 3 | 3 |
| ka1988 | 23 | 3 | 0 |
| ka817 | 81 | 10 | 2 |
| ka885 | 50 | 5 | 2 |
| ka886 | 25 | 3 | 0 |
| ka932 | 17 | 3 | 0 |
| ka967 | 6 | 0 | 0 |
| Total: 373 | 324 | 41 | 8 |
