# Supplementary material for: Selective Reduction of AMPA Currents onto Hippocampal Interneurons Impairs Network Oscillatory Activity
Source: PLoS One. 2012 Jun 4;7(6):e37318. doi: 10.1371/journal.pone.0037318 (PMC3366956; doi:10.1371/journal.pone.0037318)
Supplement: Text S1 — Supporting methods and results. (DOC) [file pone.0037318.s011.doc]

# Text S1: Supporting methods and results

**Title**: Selective Reduction of AMPA Currents onto Hippocampal Interneurons Impairs Network Oscillatory Activity

**Authors**: Antonio Caputi*^1^, Elke C. Fuchs*^1^, Kevin Allen*^1^, Corentin Le Magueresse^1^ and Hannah Monyer^#1^

*These authors contributed equally to this study

^#^ Corresponding author: h.monyer@dkfz-heidelberg.de

**Affiliations**: Department of Clinical Neurobiology at the Medical Faculty of Heidelberg University and German Cancer Research Center (DKFZ), Heidelberg, Germany

## Supporting methods

### Western blot

Crude membrane fractions were prepared from adult mice at least 6 weeks after AAV-Cre injections. Brain areas were dissected, homogenized in buffer A (0.32 M sucrose, 10 mM HEPES, pH 7.4 plus Complete protease inhibitors; Roche, Mannheim, Germany), and centrifugated at 1000 x g for 10 min. Supernatant was recovered and crude membrane fractions were pelleted at 20,000 x g for 20 min. The pellet was resuspended in buffer B (50 mM Tris, pH 7.4, 150 mM NaCl, 50 mM KCl, 1 % Triton X-100 plus Complete protease inhibitors), incubated at 4°C for 45 min and subjected to centrifugation for 10 min at 20,000 x g. Supernatant was recovered and protein concentration was measured using the Bradford method (Bio-Rad, Munich, Germany). Membrane fractions were analyzed by semi-quantitative Western blot. For immunoblot analysis protein, samples were boiled in SDS gel Laemli buffer. 20 mg of denatured crude membrane proteins were separated by SDS-PAGE, transferred onto PVDF membranes (Mini-PROTEAN Electrophoresis System; Mini trans blot; Immun-Blot PVDF Membrane; Bio-Rad, Munich Germany) and probed with polyclonal GluA1 (1:1000); GluA2/3 (1:3000); GluA4 (1:200, all GluA antibodies obtained from Millipore, Temecula, CA), GluN1 (1: 250, Invitrogen, Darmstadt, Germany); actin (1:40000); synapsin (1:5000, both Sigma, Saint Louis, MO). Peroxidase conjugated antibody (Vector, Burlingame, CA) and Amersham ECL plus Western Blotting Detection Reagents (GE Healtcare Life Sciences, Freiburg, Germany) were used for blot development. Semi-quantitative evaluation was performed using ImageJ.

### Global remapping between environments

Remapping across exploratory trials was quantified by assessing the firing association between pairs of pyramidal cells recorded simultaneously. For each exploratory trial, firing rate map similarity and an instantaneous firing rate association were calculated for each cell pair. The firing rate map similarity was calculated by performing a correlation between the firing rate values of the two spatial maps. The instantaneous firing rate association was obtained by calculating the firing rate of the two cells during 100 ms time windows and performing a correlation between the two instantaneous firing rate vectors. Remapping was assessed by performing a correlation between the firing rate map similarity scores during two trials in different environments. To control for the stability of the spatial representation across time, a correlation was performed between the firing rate map similarity scores during two trials in the same environment. Correlation between instantaneous firing rate associations were also performed using two trials in the different environments or two trials in the same environment.

## Supporting results

## Intact global remapping of hippocampal pyramidal cells in GluA4^HC-/-^ mice

When an animal moves across different environments, the hippocampal spatial representation undergoes global remapping, causing a reorganization of firing associations between pairs of pyramidal cells. We tested whether *GluA4^HC-/-^* mice showed normal degrees of global remapping between exploratory trials in the open field and the zigzag maze. The activity of pairs of simultaneously recorded pyramidal cells was studied. For each exploratory trial, firing rate map similarity and instantaneous firing rate association were calculated for each cell pair. At the population level, global remapping leads to a change in place map similarity and in instantaneous firing rate correlations. In contrast, when the mouse visit the same environment twice, the activation of the same spatial representation should lead to very similar map similarity and instantaneous firing rate associations.

The correlation coefficients between map similarity scores are shown in Supporting Figure 8*A*. Global remapping was observed for pyramidal cells in *GluA4^HC-/-^* and control mice. Map similarity scores of cell pairs were highly correlated between two trials in the same environment (control *n* = 5292 cell pairs, *r* = 0.53, *p* < 10^-16^, *GluA4^HC-/-^ n* = 2619 cell pairs, *r* = 0.56, *p* < 10^-16^, difference between genotypes: *p* = 0.0431). In contrast, the correlations between map similarity scores in different environments were approaching 0 in cell pairs from both genotypes (control *r* = 0.024, *p* = 0.08, *GluA4^HC-/-^ r* = 0.001, *p* = 0.95, difference between genotypes: *p* = 0.235). Similar results were obtained when considering instantaneous firing rate associations (Supporting Figure 8*B*).
